# Global prevalence of vancomycin-resistant *Enterococcus* in patients with haematological malignancies: a systematic review and meta-analysis

**DOI:** 10.3389/fpubh.2026.1945305

**Published:** 2026-10-15

**Authors:** Fatimah Suliman Mohammad Aljebaly

**Affiliations:** Department of Pathology, College of Medicine, Qassim University, Qassim, Saudi Arabia

**Keywords:** antibiotic resistance, epidemiology, hematological malignancy, HSCT, meta-analysis vancomycin-resistant *Enterococcus*

## Abstract

**Background:**

This systematic review and meta-analysis evaluated the global prevalence of vancomycin-resistant enterococci among patients with hematological malignancies, particularly vulnerable immunocompromised populations including leukemia, lymphoma, neutropenia, and hematopoietic stem cell transplantation patients.

**Methods:**

A systematic literature search of PubMed, Scopus, and Google Scholar was conducted for studies published up to March 2026. Eligible observational studies reporting the prevalence of VRE in patients with hematological malignancies were included. Study quality was assessed using the Joanna Briggs Institute checklist and PRISMA 2020 guidelines. Random-effects meta-analysis using logit-transformed proportions was performed in OpenMetaAnalyst and Comprehensive Meta-Analysis software. Heterogeneity was assessed using Cochran’s *Q* and *I^2^* statistics. Subgroup analysis, sensitivity analysis, publication bias assessment, and meta-regression were conducted to identify potential sources of heterogeneity.

**Results:**

A total of 120 studies involving patients with hematological malignancies were included. The pooled prevalence of VRE was 18.4% (95% CI: 15.5–21.6%), with substantial heterogeneity (*I^2^* = 97.56%, *p* < 0.001). Publication bias was detected by funnel plot asymmetry, Egger’s regression (*p* = 0.0224), and Begg’s test (*p* = 0.0226). Subgroup analysis demonstrated considerable geographic variation, with the highest prevalence observed in South America (25.5%) and Australia (22.8%). HSCT patients showed the highest pooled prevalence (97.1%), followed by neutropenic patients (91.2%) and acute myeloid leukemia patients (68.3%). Meta-regression identified continent as a significant contributor to heterogeneity (*Q* = 11.68, *p* = 0.0395), whereas detection method and sample type were not significant moderators.

**Conclusion:**

VRE represents a substantial global burden among patients with hematological malignancies, particularly in HSCT and neutropenic populations.

**Systematic review registration:**

10.17605/OSF.IO/2VH9D.

## 1 Introduction

Vancomycin-resistant enterococci (VRE) have emerged as major nosocomial pathogens and represent a growing global public health concern due to their increasing antimicrobial resistance, persistence in healthcare environments, and association with severe invasive infections. Enterococci, particularly *Enterococcus faecium* and *Enterococcus faecalis*, are commensal organisms of the human gastrointestinal tract but have evolved into important opportunistic pathogens capable of causing bloodstream infections, urinary tract infections, endocarditis, and intra-abdominal infections, especially among immunocompromised individuals (1–3). The emergence of vancomycin resistance has substantially complicated the management of enterococcal infections because treatment options are often limited, leading to prolonged hospitalization, increased healthcare costs, and elevated mortality rates (4, 5).

Among hospitalized populations, patients with hematological malignancies are particularly vulnerable to VRE colonization and infection. Hematological malignancies, including acute myeloid leukemia (AML), acute lymphoblastic leukemia (ALL), lymphoma, multiple myeloma, and myelodysplastic syndromes, are commonly associated with profound immunosuppression resulting from both the underlying disease and intensive chemotherapy regimens. Additionally, these patients frequently experience prolonged neutropenia, repeated hospital admissions, invasive procedures, mucosal barrier injury, and extensive exposure to broad-spectrum antibiotics, all of which contribute to the acquisition and dissemination of multidrug-resistant organisms such as VRE (6, 7).

The epidemiology of VRE has changed dramatically over the past two decades. Initially recognized in Europe and the United States, VRE has now spread globally, with increasing prevalence reported across Asia, the Middle East, Africa, and South America (8, 9). The widespread use of glycopeptides, cephalosporins, carbapenems, and other broad-spectrum antimicrobial agents has accelerated the selection and persistence of resistant enterococcal strains within healthcare settings. Hospital environments, contaminated medical equipment, healthcare workers’ hands, and colonized patients serve as important reservoirs for transmission (10). Furthermore, *E. faecium* has demonstrated remarkable adaptability through the acquisition of resistance genes such as *vanA* and *vanB*, which confer high-level resistance to vancomycin and significantly limit therapeutic options (1, 11).

Patients with hematological malignancies represent one of the highest-risk groups for VRE colonization and subsequent bloodstream infection. Gastrointestinal colonization often precedes invasive infection, particularly during periods of severe neutropenia or mucosal injury following chemotherapy or hematopoietic stem cell transplantation (HSCT). Previous studies have demonstrated that VRE colonization significantly increases the risk of subsequent bacteremia, septic complications, and mortality among leukemia and HSCT patients. Moreover, VRE bloodstream infections have been associated with delayed engraftment, prolonged intensive care unit stay, increased use of last-line antibiotics such as linezolid and daptomycin, and poorer clinical outcomes (12–14).

HSCT recipients are especially susceptible to VRE due to intensive immunosuppressive therapy, prolonged hospitalization, disruption of intestinal microbiota, and repeated antimicrobial exposure. Recent evidence suggests that pre-transplant gastrointestinal colonization with VRE is an important predictor of post-transplant bloodstream infection. A recent global systematic review and meta-analysis involving HSCT recipients reported a substantial burden of VRE infection worldwide and highlighted considerable regional variability in prevalence estimates (12). However, despite increasing recognition of VRE in HSCT settings, there remains limited comprehensive evidence synthesizing the prevalence of VRE across the broader spectrum of hematological malignancies.

The increasing burden of antimicrobial resistance among enterococci poses major clinical and infection control challenges. VRE strains are often resistant to multiple antibiotic classes, including aminoglycosides, β-lactams, and fluoroquinolones, thereby reducing effective therapeutic options. The emergence of daptomycin-resistant and linezolid-resistant VRE strains further complicates treatment strategies. Recent molecular epidemiological studies have demonstrated the persistence and horizontal transmission of highly resistant VRE clones within oncology and transplant units, emphasizing the importance of active surveillance and infection prevention measures (15, 16).

Accurate estimates of VRE prevalence among hematological malignancy patients are essential for informing antimicrobial stewardship programs, guiding empirical therapy, improving infection prevention strategies, and supporting policy development. However, published studies have reported highly variable prevalence rates due to differences in geographic region, patient populations, diagnostic methods, sample types, healthcare practices, and study design. Some studies focus primarily on colonization and HSCT, whereas others report invasive bloodstream infections, leading to substantial heterogeneity in reported outcomes. In addition, laboratory methods differ in sensitivity: culture-based methods are specific but relatively slow and less sensitive; automated systems improve standardized detection and sensitivity; molecular assays generally offer the highest sensitivity and rapid detection; while chromogenic media provide rapid, inexpensive species identification but may have variable sensitivity. Although several primary studies and narrative reviews have explored VRE epidemiology in hematological patients, there remains a lack of comprehensive pooled evidence evaluating the global prevalence of VRE across different hematological malignancies and clinical settings. Systematic review and meta-analysis provide robust approaches for synthesizing available evidence, quantifying pooled prevalence estimates, and identifying potential sources of heterogeneity among studies. Such analyses are particularly important in the context of emerging antimicrobial resistance and the increasing clinical burden of multidrug-resistant infections in immunocompromised populations.

Therefore, the present systematic review and meta-analysis aimed to estimate the pooled prevalence of VRE among patients with hematological malignancies globally. Additionally, subgroup analyses and meta-regression were conducted to evaluate the influence of geographic region, hematological malignancy type, diagnostic method, and sample type on prevalence estimates and study heterogeneity. The findings of this study are expected to provide valuable epidemiological evidence to support infection prevention strategies, antimicrobial stewardship interventions, and future research targeting VRE among vulnerable hematological populations.

## 2 Materials and methods

### 2.1 Study design and protocol

For this study, the Preferred Reporting Items for Systematic Reviews and Meta-Analysis (PRISMA 2020) guidelines (17) was used as the checklist. This study protocol has been registered on the Open Science Framework (OSF) with the identification 10.17605/OSF.IO/2VH9D

### 2.2 Literature review

Abstracts from PROSPERO database and database of abstracts of reviews of effects (DARE) (http://www.library.UCSF.edu) were searched to ensure that no other meta-analysis on the prevalence of VRE in patients with hematological malignancies exists or is ongoing. PubMed, Google scholar and Scopus were methodically explored for peer-reviewed articles published, which had the relevant study populations (clinical or community surveys), study design and expected outcomes for this review. These databases were searched on 5th March, 2026 using the search strategy in Pubmed; ((“vancomycin-resistant enterococci”[MeSH Terms] OR “vancomycin-resistant enterococcus”[Title/Abstract] OR VRE[Title/Abstract]) AND (“hematologic neoplasms”[MeSH Terms] OR “hematological malignancies”[Title/Abstract] OR leukemia[Title/Abstract] OR lymphoma[Title/Abstract] OR myeloma[Title/Abstract] OR “hematopoietic stem cell transplantation”[Title/Abstract] OR HSCT[Title/Abstract] OR neutropenia[Title/Abstract]) AND (prevalence[Title/Abstract] OR epidemiology[Title/Abstract] OR colonization[Title/Abstract] OR carriage[Title/Abstract] OR infection[Title/Abstract] OR surveillance[Title/Abstract])). = 6,695 and in Google scholar; prevalence of VRE in Hematology patients = 5,720. Scopus; TITLE-ABS-KEY ((“vancomycin-resistant enterococci” OR VRE) AND (“hematological malignanc*” OR leukemia OR lymphoma OR myeloma OR HSCT) AND (prevalence OR epidemiology OR colonization)) = 195. In addition, references and titles from included articles were utilized as a supplementary search tool.

### 2.3 Outcome definition, inclusion and exclusion criteria for studies

#### 2.3.1 Outcome definition

The primary outcome of this systematic review was the prevalence of VRE detection among patients with hematological malignancies. Because the included studies differed substantially in the clinical context and specimen type used for VRE detection, outcomes were further categorized into clinically relevant groups.

Studies using stool or rectal-swab specimens for surveillance or microbiological screening were classified as VRE colonization/carriage, representing gastrointestinal carriage of VRE. Studies using blood specimens were classified as bloodstream VRE detection, reflecting recovery or detection of VRE from blood and representing a more invasive clinical context. However, because clinical diagnostic criteria for bloodstream infection were not consistently reported across the included studies, positive blood cultures were not automatically interpreted as clinically confirmed VRE infection.

For the primary analysis, all eligible studies reporting quantitative VRE detection data were included to estimate the overall prevalence of VRE detection. Pre specified outcome-stratified analyses were subsequently performed for (i) VRE colonization/carriage based on stool or rectal-swab specimens and (ii) bloodstream VRE detection based on blood specimens. Where studies reported sufficient information to distinguish these outcomes, the outcome definition used by the original investigators was retained.

#### 2.3.2 Inclusion criteria

Studies were considered eligible for inclusion if they; were carried out in the hematological unit; involved patients diagnosed with hematological malignancies, including leukemia, lymphoma, multiple myeloma, myelodysplastic syndromes, or other hematologic cancers, included HSCT or bone marrow transplant recipients with underlying hematological malignancies; reported the prevalence, incidence, colonization, carriage, or infection rate of VRE; identified VRE isolates using standard microbiological culture, antimicrobial susceptibility testing, or molecular diagnostic methods; and employed observational study designs, including cross-sectional, cohort, case–control, or surveillance studies. Furthermore, studies were required to provide sufficient quantitative data to enable extraction or calculation of VRE prevalence estimates.

#### 2.3.3 Exclusion criteria

Studies were excluded if they; were not carried out in the hematological unit; involved patients with solid tumors only without separate data for hematological malignancies; focused exclusively on environmental, veterinary, or non-human samples; did not specifically report vancomycin resistance among *Enterococcus* species; were reviews, systematic reviews, meta-analyses, editorials, commentaries, conference abstracts without full texts, letters, or case reports with insufficient sample size; contained duplicate or overlapping datasets, in which case the most comprehensive or recent publication was retained; or lacked sufficient data for extraction and meta-analysis. Articles published in languages other than English or studies with inaccessible full texts were also excluded.

### 2.4 Data extraction

Study selection and data extraction were conducted using a predefined, standardized protocol developed prior to literature screening. Titles and abstracts were initially screened for eligibility, followed by full-text assessment of potentially relevant articles. To minimize selection bias and extraction errors, each screening and data extraction step was conducted independently at two separate time points by the reviewer, with discrepancies resolved through repeated verification. Data extraction was performed using a piloted standardized extraction form capturing the first author’s name, publication year, the country and continent where study was carried out, characteristics of the population group where VRE were detected, the type of hematological malignancies, the type of detection method used, and the type of sample collected.

Following removal of duplicate records, the retrieved citations were imported into Rayyan for screening. Title and abstract screening was performed independently by the designated reviewers using the predefined inclusion and exclusion criteria. Records considered potentially eligible were retrieved for full-text assessment.

Full-text articles were independently assessed by the reviewers against the predefined eligibility criteria. Reviewers worked independently during the screening process, and their decisions were recorded separately in Rayyan. Disagreements between reviewers were resolved through discussion and consensus. When consensus could not be reached, a third reviewer was consulted and the final decision was made following adjudication.

The three independent reviewers invited through Rayyan (Qatar Computing Research Institute) cross-checked the selections but were not involved in manuscript drafting and preparations.

### 2.5 Study quality assessment

The Joanna Briggs Institute (JBI) critical appraisal checklist for prevalence data (JBI) (Supplementary file 1) was used to evaluated quality of the included studies. This verification approach ensured methodological consistency, reproducibility, and data integrity. The JBI checklist was used for item-level critical appraisal. Studies were not assigned an overall score; instead, risk of bias was interpreted across domains. This appraisal checklist contains nine items. Item grades are yes, no, unclear, or not applicable (JBI). Studies were included in the analysis if consensus was reached among two reviewers (145). The quality of the 120 included studies is given in (Supplementary file 2).

### 2.6 Data analysis

The primary meta-analysis estimated the overall pooled prevalence of VRE detection among patients with hematological malignancies. To account for clinical heterogeneity in the reported outcomes, additional subgroup meta-analyses were performed according to outcome category. Studies using stool or rectal-swab specimens were analyzed as VRE colonization/carriage, whereas studies using blood specimens were analyzed as bloodstream VRE detection. These categories were not interpreted as equivalent clinical outcomes.

Random-effects meta-analysis using logit-transformed proportions was applied because substantial between-study heterogeneity was anticipated. Pooled estimates were reported with 95% confidence intervals. Additional subgroup analyses were conducted according to geographical region, hematological malignancy and detection method. Heterogeneity was assessed using Cochran’s *Q* and *I^2^* statistics. DerSimonian and Laird method of meta-analysis (18, 19) was used to determine the pooled prevalence.

### 2.7 Bias and heterogeneity analysis

Within-study biases were evaluated according to the country and continent where study was carried out, type of hematological malignancies, the detection method used and the type of sample collected. Small study effects or bias were examined by funnel plots and trim-and-fill plots. The heterogeneities of study-level estimates were assessed by Cochran’s *Q* test. The percentage of the variation in prevalence estimates attributable to heterogeneity was measured by the inverse variance index (*I*^2^), and *I^2^* values of 25, 50, and 75% were considered low, moderate, and high heterogeneity, respectively (19). The sources of heterogeneity were analyzed using the sensitivity analysis (leave-one-out meta-analysis), subgroup analysis, and meta-regression. Meta-analysis was performed using OpenMeta Analyst software (20) and Comprehensive meta-analysis version 3 (CMA) (144).

## 3 Results

### 3.1 Search results and eligible studies

Through database searches, a total of 12,610 studies were found, with 7,000 duplicates deleted. The titles and abstracts of the remaining 5,610 papers were examined, and 5,455 were excluded after failing to match any of the inclusion criteria. A total of 155 full-text papers were reviewed for eligibility, with 35 excluded for various reasons (Figure 1). A total of 120 full-text studies were used for qualitative and quantitative analyses (Figure 1). The characteristics of the included studies are shown in Table 1 and the characteristics of hematological malignancies as reported by different studies is shown in Supplementary Table 1.

**Figure 1:**
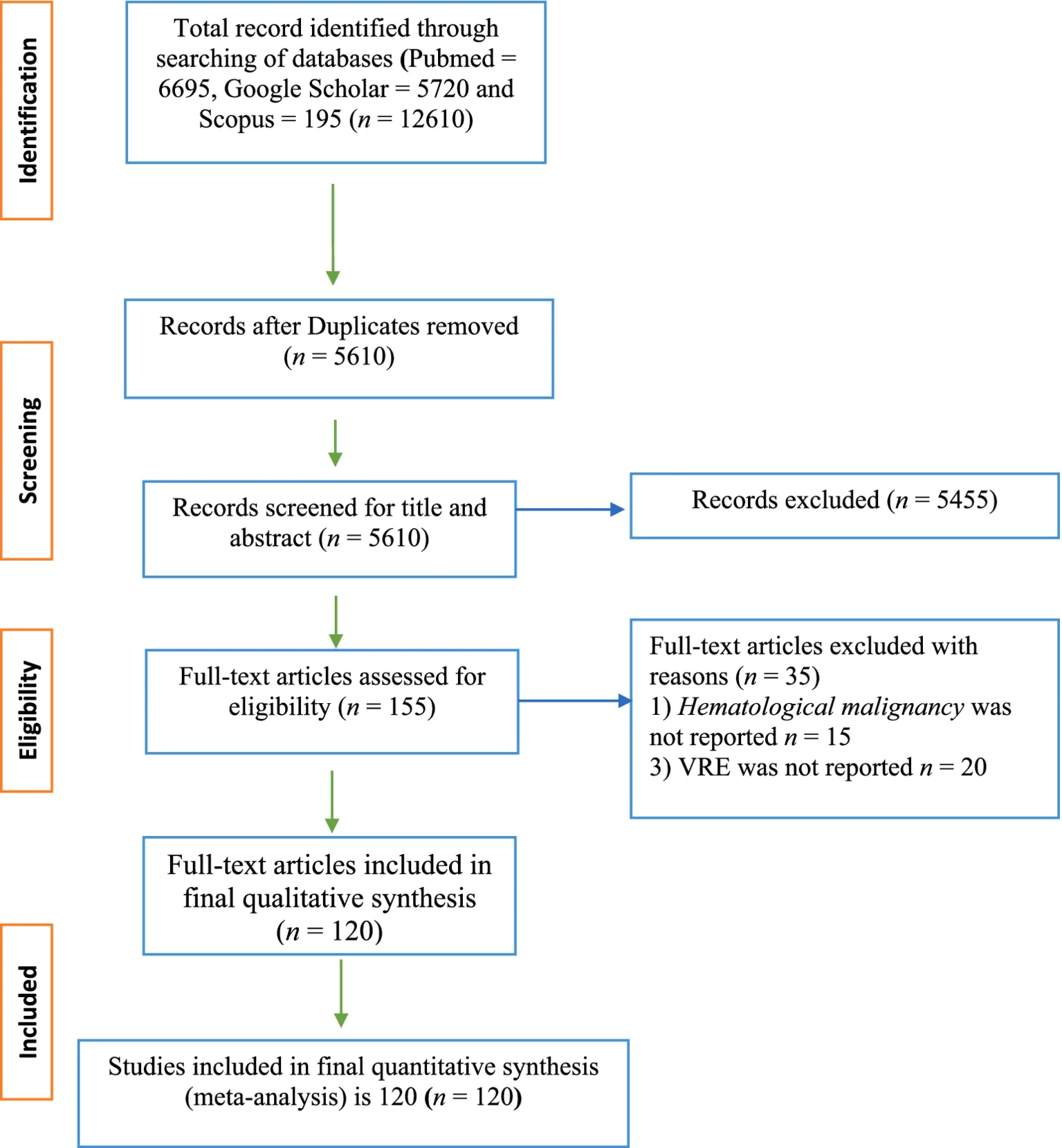
PRISMA flow diagram for the selection of eligible articles included in the study.

**Table 1:** Characteristics of the included studies reporting the prevalence of VRE in patients with hematological malignancies.

S/NO	Author, Publication year	Country	Continent	Detection method	Sample type	Haematological Malignancies	Sample Size	Number positive
1	Suntharam et al. (2002) (26)	USA	North America	Agar dilution	Blood	AML (12)	155	12
2	Liss et al. (2012) (27)	Germany	Europe	E test	Stool	AML (51)	513	51
3	Gedik et al. (2014) (28)	Turkiye	Europe	E test	Blood	AML (39), ALL (9), MM (1), NHL (1)	126	50
4	Hefazi et al. (2016) (29)	USA	North America	Agar dilution	Blood	AML (96)	203	96
5	Mioljevi et al. (2013) (30)	Serbia	Europe	Disk diffusion	Stool	AML (19), ALL (5), NHL (1), CLL (2)	65	27
6	Chhatwal et al. (2020) (31)	Germany	Europe	VITEK 2	Stool	HSCT (132)	555	132
7	Cattaneo et al. (2018) (32)	Italy	Europe	Broth microdilution	Rectal swab	AML (4), Lymphoma (5)	144	9
8	Ford et al. (2015) (33)	USA	North America	Microscan	Stool	HSCT (224)	508	224
9	Ford et al. (2015) (34)	USA	North America	Microscan	Stool	AML (61), ALL (21)	214	82
10	Taur et al. (2012) (35)	USA	North America	PCR	Stool	AML (35)	94	35
11	Kamboj et al. (2010) (36)	USA	North America	Disk diffusion	Rectal swab	HSCT (68)	247	68
12	Calderwood et al. (2008) (37)	USA	North America	Disk diffusion	Rectal swab	HSCT (134)	416	134
13	Weinstock et al. (2007) (38)	USA	North America	Microscan	Stool	AML (37)	92	37
14	Matar et al. (2006) (39)	USA	North America	Disk diffusion	Rectal swab	AML (56), HSCT (32), Lymphoma (11)	2,115	99
15	Tsiatis et al. (2004) (40)	USA	North America	Broth microdilution	Stool	AML(6), ALL(4), MDS(1), ES(1), HL(1), HYP(1), WAS(1)	61	15
16	Shaikh et al. (2002) (41)	USA	North America	Broth microdilution	Rectal swab	AML(4)	39	4
17	Henning et al. (1996) (42)	USA	North America	E test	Stool	Tumor (23)	73	23
18	Montecalvo et al. (1995) (43)	USA	North America	E test	Rectal swab	Tumor (86)	306	86
19	Rubin et al. (1992) (44)	USA	North America	E test	Rectal swab	Tumor (4)	26	4
20	Levitt et al. (2014) (45)	USA	North America	E test	Rectal swab	AML (65), HSCT (33)	312	98
21	Song et al. (2016) (46)	USA	North America	E test	Rectal swab	HSCT (3)	44	3
22	Zirakzadeh et al. (2007) (47)	USA	North America	E test	Stool	AML (13), MDS (4), ALL (3), CLL (1)	217	22
23	Embil et al. (1996) (48)	Canada	North America	Disk diffusion	Stool	HSCT (0)	30	0
24	Thacker et al. (2014) (49)	India	Asia	E test	Stool	HSCT (65)	618	65
25	Nateghian et al. (2011) (50)	Iran	Asia	PCR	Stool	ALL (33)	130	33
26	Aktürk et al. (2016) (51)	Turkiye	Europe	E test	Rectal swab	ALL (35), AML (18), Tumor (19)	229	72
27	Devrim et al. (2015) (52)	Turkiye	Europe	E test	Rectal swab	ALL (18), AML (4), HL (2)	120	24
28	Bradley et al. (2002) (53)	UK	Europe	E test	Stool	HSCT (120)	293	120
29	Gray and George (2000) (54)	UK	Europe	E test	Stool	ALL (59)	154	59
30	Nourse et al. (2000) (55)	Ireland	Europe	PCR	Stool	HSCT (4)	137	4
31	Guiot et al. (1991) (56)	Netherlands	Europe	Disk diffusion	Stool	HSCT (9)	95	9
32	Schuster et al. (1998) (57)	Germany	Europe	Disk diffusion	Stool	HSCT (24)	48	24
33	Plessis et al. (1995) (58)	France	Europe	Disk diffusion	Stool	HSCT (14)`	132	14
34	Bell et al. (2013) (59)	Australia	Australia	PCR	Rectal swab	HSCT (18)	229	18
35	Padiglione et al. (2003) (60)	Australia	Australia	PCR	Rectal swab	HSCT (4)	285	4
36	Arman et al. (2022) (61)	Palestine	Asia	Disk diffusion	Blood	HSCT (9)	57	9
37	Stoeva et al. (2025) (62)	Bulgaria	Europe	Disk diffusion	Blood	HSCT (45)	180	45
38	Xie et al. (2020) (63)	Australia	Australia	Chrom agar	Rectal swab	AML (49)	72	49
39	Worth et al. (2007) (64)	Australia	Australia	E test	Blood	AML (9), DLBL (1), NHL (1), MM (1), ES (1), APML (1)	41	14
40	Roghmann et al. (1997) (65)	USA	North America	E test	Blood	ALL (33)	51	33
41	Oh et al. (2004) (66)	South Korea	Asia	E test	Stool	AML (14), Anemia (1), MM (1), Lymphoma (1)	82	17
42	Gambarotto et al. (2000) (67)	France	Europe	E test	Rectal swab	Neutropenia (44)	70	44
43	Carvalho et al. (2020) (68)	Australia	Australia	E test	Blood	AML (25)	42	25
44	Niyazi et al. (2024) (69)	Bulgaria	Europe	Broth Microdilution	Stool	HSCT (15)	48	15
45	Webb et al. (2020) (70)	USA	North America	E test	Stool	Neutropenia (480)	1,578	480
46	Kirby et al. (2006) (71)	USA	North America	Broth microdilution	Blood	Neutropenia (88)	404	88
47	Hristova et al. (2023) (72)	Bulgaria	Europe	E test	Stool	MDS (6), MM (7), CLL (3), AML (2), NHL (1)	119	19
48	Ümit et al. (2016) (73)	Turkiye	Europe	Disk diffusion	Rectal swab	AML (25), ALL (7), CLL (3), MM (11), Lymphoma (12), CML (1), MDS (5), Tumor (2),	178	66
49	Chen et al. (2017) (74)	Taiwan	Asia	Phoenix system	Blood	Neutropenia (55)	118	55
50	Kara et al. (2015) (75)	Turkiye	Europe	Phoenix system	Blood	Neutropenia (2)	32	2
51	Bajpai et al. (2023) (76)	India	Asia	Disk diffusion	Blood	Neutropenia (1)	19	1
52	Choi et al. (2022) (77)	South Korea	Asia	VITEK 2	Blood	AML (4), ALL (30), MM (1)	72	35
53	Krull et al. (2016) (78)	Germany	Europe	VITEK 2	Rectal swab	Lymphoma (1), MM (1), AML (11), ALL (1), MDS (3), Histiocystosis (1), AA (1), ARM (1)	394	20
54	Sabir et al. (2021) (79)	Pakistan	Asia	Disk diffusion	Blood	HSCT (67)	246	67
55	Chen et al. (2021) (80)	China	Asia	VITEK 2	Blood	Neutropenia (0)	15	0
56	Heisel et al. (2017) (81)	USA	North America	Chrom agar	Rectal swab	AML (123), MDS (11)	229	134
57	Alatorre-Fernández et al. (2017) (82)	Mexico	North America	VITEK 2	Blood	ALL (17), Lymphoma (5), MDS (1)	568	23
58	Ozorowski et al. (2009) (83)	Poland	Europe	Disk diffusion	Blood	ALL (1)	10	1
59	Mekonnen et al. (2025) (84)	Ethiopia	Africa	E test	Blood	Neutropenia (2)	125	2
60	Kirdar et al. (2010) (85)	Turkiye	Europe	Phoenix system	Rectal swab	Neutropenia (12)	12	12
61	Klyasova et al. (2018) (86)	Russia	Europe	Broth microdilution	Blood	HSCT (52)	427	52
62	Rattanon et al. (2025) (87)	Thailand	Asia	Sensititre	Rectal swab	AML (3), ALL (1)	53	4
63	Fedorova et al. (2025) (25)	Russia	Europe	Broth microdilution	Blood	HSCT (122)	465	122
64	Leis et al. (2006) (88)	USA	North America	Broth microdilution	Stool	ALL (16), NHL (11), MDS (4)	189	31
65	Torres et al. (2022) (89)	Spain	Europe	Broth microdilution	Rectal swab	AML (9)	149	9
66	Braak et al. (2000) (90)	Netherlands	Europe	PCR	Stool	HSCT (15)	1,112	15
67	Sengar et al. (2014) (91)	India	Asia	E test	Blood	Neutropenia (46)	242	46
68	Wang et al. (2015) (92)	China	Asia	Disk diffusion	Blood	HSCT (1)	29	1
69	de la Cruz-Hernández et al. (2020) (93)	Mexico	North America	Disk diffusion	Blood	Neutropenia (1)	202	1
70	Mattei et al. (2022) (94)	Italy	Europe	Chrom agar	Blood	Neutropenia (0)	97	0
71	Kaveh et al. (2016) (95)	Iran	Asia	E test	Stool	ALL (6), HL (1), NHL (3), MM (2), IDS (1), SCID (1)	42	14
72	Ahmad et al. (2024) (96)	Iraq	Asia	VITEK 2	Blood	HSCT (13)	26	13
73	Chiara Cattaneo et al. (2008) (97)	Italy	Europe	Disk diffusion	Blood	ALL (3)	13	3
74	Hackney et al. (2019) (98)	USA	North America	Disk diffusion	Blood	AML (42)	77	42
75	Ali et al. (2018) (99)	Ethiopia	Africa	Disk diffusion	Stool	IDS (7)	112	7
76	Kara et al. (2015) (100)	Turkiye	Europe	Disk diffusion	Rectal swab	IDS (1), AML (1), ALL (1)	193	3
77	Hung et al. (2021) (101)	Taiwan	Asia	Agar dilution	Stool	Neutropenia (66)	314	66
78	Amanati et al. (2021) (102)	Iran	Asia	Disk diffusion	Blood	Neutropenia (2)	152	2
79	Knoll et al. (2005) (103)	Germany	Europe	Broth microdilution	Stool	Neutropenia (44)	1,124	44
80	Scheich et al. (2018) (104)	Germany	Europe	VITEK 2	Blood	AML (122)	264	122
81	Satlin et al. (2014) (105)	USA	North America	VITEK 2	Blood	HSCT (56)	352	56
82	Joudeh et al. (2023) (106)	Palestine	Asia	VITEK 2	Blood	Neutropenia (2)	9	2
83	MacAllister et al. (2018) (107)	USA	North America	Chrom agar	Rectal swab	HSCT (90)	1,492	90
84	Teferi et al. (2025) (108)	Ethiopia	Africa	Disk diffusion	Stool	ALL (4), NHL (3), Tumor (1), AML (3)	87	11
85	Çalişkan Demirkiran et al. (2026) (109)	Turkiye	Europe	Disk diffusion	Blood	HSCT (27)	118	27
86	Khateb et al. (2025) (110)	Saudi Arabia	Asia	E test	Blood	HSCT (23)	47	23
87	Ali et al. (2006) (111)	Egypt	Africa	Broth microdilution	Stool	Neutropenia (6)	50	6
88	Kim and Choi (2022) (112)	South Korea	Asia	E test	Blood	ALL (8), AML (33), MDS (9), AA (4), NHL (14), MM (9)	232	77
89	Mendes et al. (2020) (113)	Brazil	South America	VITEK 2	Blood	HSCT (53)	413	53
90	Park et al. (2024) (114)	South Korea	Asia	E test	Blood	AML (12), ALL (4), MM (2)	328	18
91	Yu et al. (2015) (115)	China	Asia	E test	Blood	ALL (5), CML (3)	125	8
92	Ornstein et al. (2015) (116)	USA	North America	VITEK 2	Blood	AML (37)	350	37
93	Sadowska-Klasa et al. (2017) (117)	Poland	Europe	E test	Rectal swab	HSCT (37)	120	37
94	Battipaglia et al. (2019) (118)	Italy	Europe	E test	Rectal swab	AML (1), ALL (1)	10	2
95	Vydra et al. (2012) (119)	USA	North America	E test	Blood	HSCT (173)	752	173
96	Dragičević-Jojkić et al. (2021) (120)	Serbia	Europe	Disk diffusion	Blood	Neutropenia (7)	17	7
97	Navarra et al. (2025) (121)	Italy	Europe	PCR	Rectal swab	HSCT (1037)	2,101	1,037
98	Fanci et al. (1999) (122)	Italy	Europe	E test	Blood	AML (1)	11	1
99	Zajac-Spychala et al. (2021) (123)	Poland	Europe	Disk diffusion	Blood	ALL (50), AML (15), HL (4), NHL (2)	143	71
100	El Omri et al. (2024) (124)	Qatar	Asia	Phoenix system	Blood	Neutropenia (2)	9	2
101	Mokhtar et al. (2025) (8)	Saudi Arabia	Asia	VITEK 2	Blood	Neutropenia (67)	254	67
102	Baier et al. (2019) (125)	Germany	Europe	VITEK 2	Blood	HSCT (4)	71	4
103	Papanicolaou et al. (2019) (126)	USA	North America	VITEK 2	Blood	AML (207), MDS (51)	7,128	258
104	Iosifidis et al. (2012) (127)	Greece	Europe	VITEK 2	Stool	HSCT (18)	26	18
105	Dubberke et al. (2006) (128)	USA	North America	E test	Blood	AML (32), CML (5), Lymphoma (13), MDS (4), MM (2), AA (2), TC (1), OC (1)	334	60
106	Alotaibi and Bukhari (2017) (129)	Saudi Arabia	Asia	Disk diffusion	Blood	AML (2), MM (2), Neutropenia (8)	231	12
107	Marchi et al. (2018) (130)	Brazil	South America	Broth microdilution	Rectal swab	AML (22), ALL (9), CML (2), Lymphoma (1)	76	34
108	Thacker et al. (2014) (131)	India	Asia	E test	Blood	Neutropenia (2)	9	2
109	Mandal et al. (2024) (132)	India	Asia	Disk diffusion	Blood	Neutropenia (3)	6	3
110	Hijazi et al. (2009) (133)	Palestine	Asia	Disk diffusion	Stool	HSCT (19)	32	19
111	Mellouli et al. (2021) (134)	Tunisia	Africa	Broth microdilution	Blood	HSCT (2)	816	2
112	Doganci et al. (2023) (135)	Turkiye	Europe	E test	Rectal swab	ALL (5)	101	5
113	Nowicka and Gil (2026) (136)	Poland	Europe	Disk diffusion	Rectal swab	HSCT (2)	14	2
114	Colaneri et al. (2025) (137)	Italy	Europe	Chrom agar	Rectal swab	HSCT (304)	1,056	304
115	Guarnera et al. (2023) (138)	Italy	Europe	E test	Blood	AML (8)	132	8
116	Janjusevic et al. (2021) (139)	Serbia	Europe	Chrom agar	Stool	HSCT (28)	130	28
117	Cento et al. (2025) (140)	Italy	Europe	Phoenix system	Blood	HSCT (7)	992	7
118	Kampmeier et al. (2017) (141)	Germany	Europe	VITEK 2	Rectal swab	HSCT (12)	29	12
119	Kim et al. (2003) (142)	South Korea	Asia	Agar dilution	Rectal swab	HSCT (230)	452	230
120	Thomas et al. (2007) (143)	USA	North America	E test	Blood	HSCT (61)	442	61

AML, acute myeloid leukemia; CML, chronic myeloid leukemia; ALL, acute lymphocytic leukemia; CLL, chronic lymphocytic leukemia; HSCT, hematopoietic stem cell transplant; MM, multiple myeloma; MDS, myelodysplastic syndrome; HL, Hodgkin’s lymphoma; NHL, non-Hodgkin’s lymphoma; AA, aplastic anemia; IDS, immunodeficiency syndrome; SCID, severe combined immunodeficiency syndrome; ES, Ewing’s sarcoma; HYP, hypophosphatasia; WAS, Wiskatt-Aldrich syndrome; ARM, arthritis metothrevat; APML, acute promyelocytic leukemia; DLBL, diffuse large-cell-B-cell lymphoma; USA, United States of America; UK, United Kingdom; PCR, polymerase chain reaction; E test, epsilometer test.

#### 3.1.1 Methodological quality of included studies

Methodological quality was assessed using the nine-item JBI critical appraisal checklist for prevalence studies (Supplementary file 2), with item-level ratings for all 120 included studies presented in Supplementary file 3. Overall, most studies demonstrated adequate methodological quality across key domains, including appropriate sampling frames, valid identification of VRE using standard microbiological or molecular methods, and sufficient reporting of sample size and study setting. The majority of studies used clearly defined case definitions for VRE (culture-based or molecular confirmation) and reported detection methods with adequate detail, supporting reliability of the extracted prevalence data.

However, several domains showed more variable performance across studies. Coverage of the target population was frequently unclear, as many studies drew from single-center or selectively screened cohorts (e.g., actively surveyed HSCT or neutropenic patients) rather than consecutive or representative samples of all patients with hematological malignancies, raising the potential for selection bias. Similarly, standardized and reliable measurement of the condition was not consistently reported, particularly regarding clinical criteria distinguishing colonization from bloodstream infection. Response rates and completeness of data collection were adequately described in most studies, though a subset of smaller or older studies provided limited detail on these items.

No studies were excluded from the meta-analysis on the basis of JBI appraisal alone, as risk of bias was interpreted qualitatively across domains rather than through a summary score, consistent with current JBI guidance for prevalence studies. Overall, the included evidence base was judged to be of moderate-to-good methodological quality, though heterogeneity in population sampling and outcome ascertainment across studies likely contributed to the substantial statistical heterogeneity observed in the pooled analysis.

### 3.2 The overall pooled prevalence of VRE in patients with hematological malignancies

The overall pooled prevalence of VRE in patients with hematological malignancies was calculated to be 18.4% (95% CI; 15.5–21.6%; *I^2^* = 97.56%; *P* < 0.001) (Figure 2). To determine the prevalence of VRE in patients with hematological malignancies, random-effects meta-analyses were performed utilizing the total sample size and number of positives (effect size, standard error of effect size). Between-study variability was high [*t^2^* = 1.138; heterogeneity *I^2^* = 97.56% with heterogeneity chi-square (*Q*) = 4879.222, degrees of freedom (df) = 119, and *P* < 0.001]. No one study had an effect on the heterogeneity and pooled prevalence, as evidenced in the sensitivity analysis leave-one-out forest plot (Supplementary Figure 1). Furthermore, as indicated in the asymmetrical funnel plot, there was publication bias (Figure 3). In addition to the funnel plot for visual detection of publication bias, the Trim-and-Fill method was applied to include the “missing” studies from the analysis. The asymmetric studies were trimmed to detect the unbiased effect and the plot was filled by re-inserting the trimmed studies, along with their imputed counterparts. As a result, 13 studies were missed and fall at the right side of the pooled estimate (Figure 4). In the Trim-and-Fill method, the adjusted estimate of VRE in patients with hematological malignancies under the random effect model was 22.5% (95% CI; 19.1–26.2%). The Egger’s regression *t* = 2.3137 [intercept = −2.2089; 95% CI; (−4.10 – -0.32); *P* = 0.0224] and Begg’s rank correlation test (*P* = 0.0226) confirmed publication bias.

**Figure 2:**
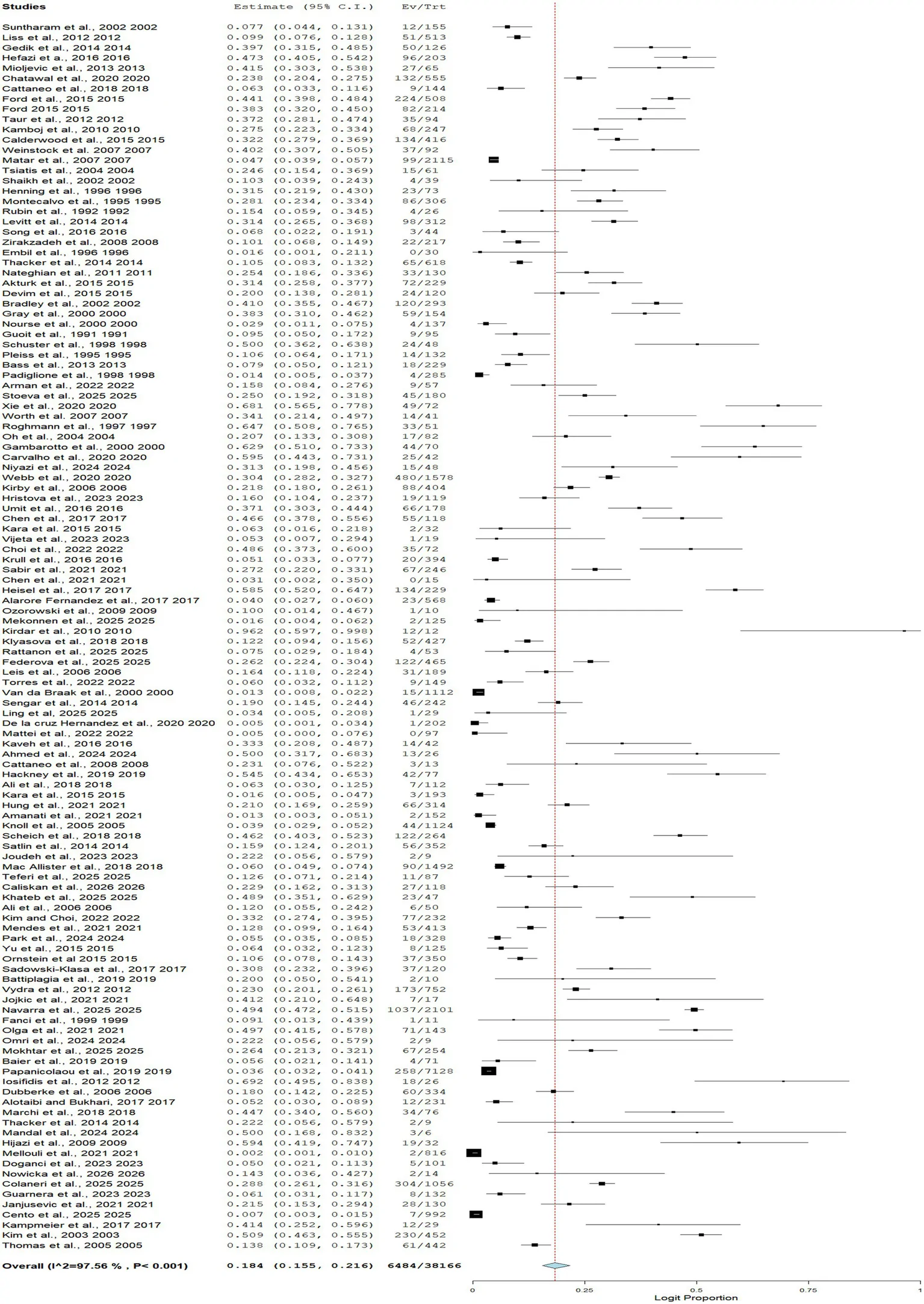
Forest plot showing the pooled prevalence of VRE in patients with hematological malignancies.

**Figure 3:**
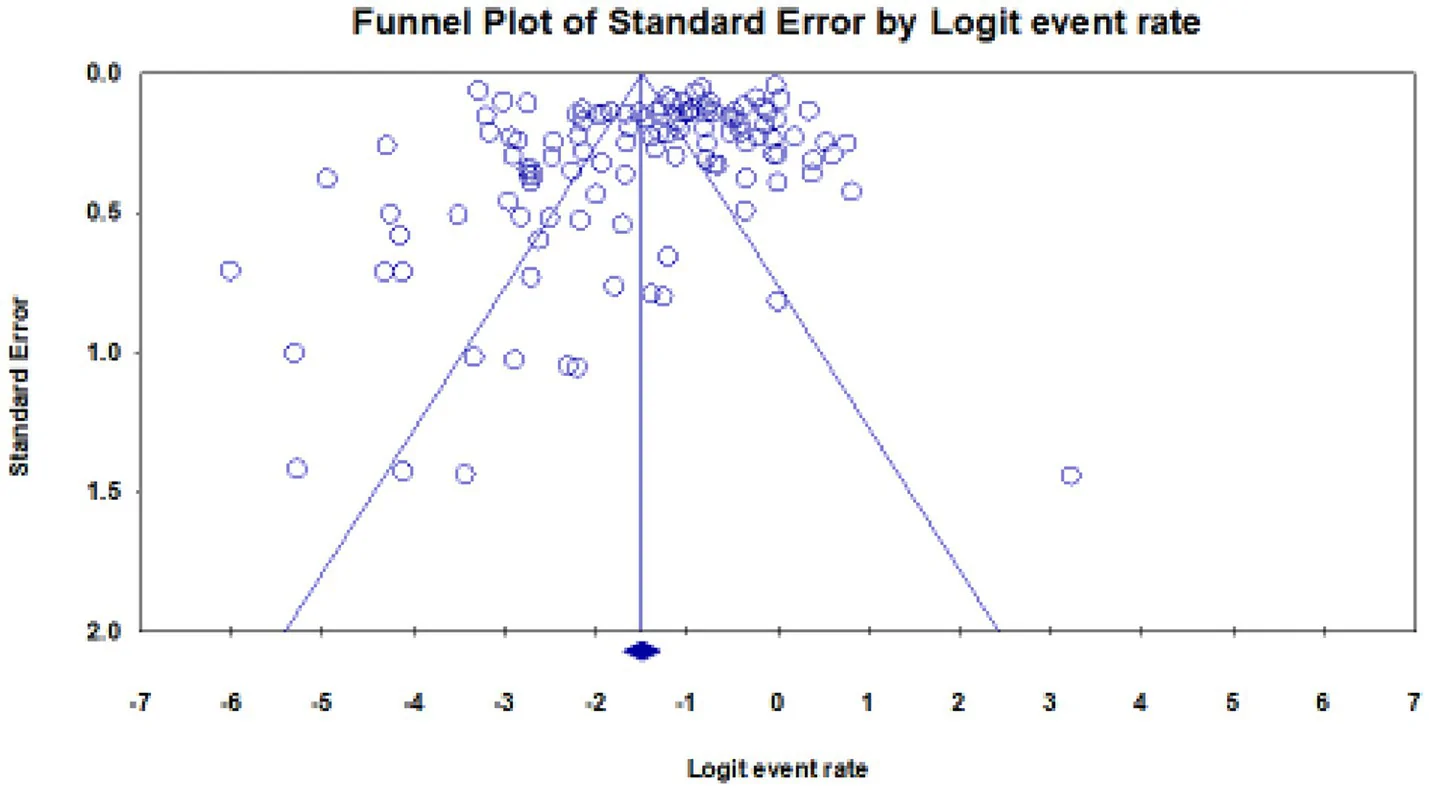
Funnel plot showing publication bias in studies reporting the prevalence of VRE in patients with hematological malignancies.

**Figure 4:**
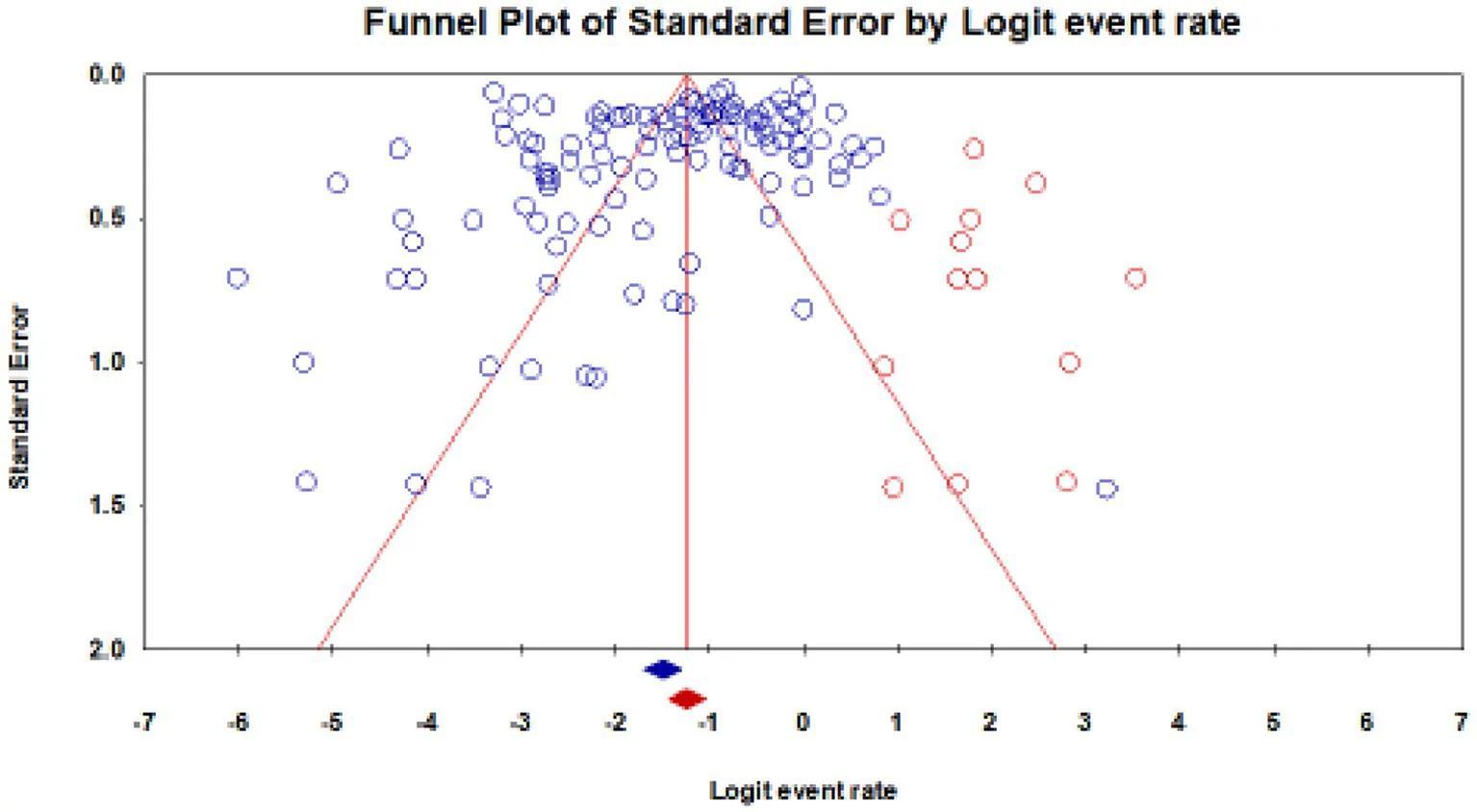
Funnel plot showing 13 added studies (in Red) in the Trim-and- Fill method reporting the prevalence of VRE in patients with hematological malignancies.

### 3.3 Subgroup meta-analysis

Subgroup analyses were performed according to the country and continent where the study was carried out, the type of hematological malignancy, the detection method used and the sample type, to find the likely sources of variability among studies, as high heterogeneity was detected (*I^2^* = 97.56%). Table 2 shows the result of subgroup analysis.

**Table 2:** Subgroup analyses of the prevalence of VRE in patients with hematological malignancies.

Outcome specific	Number of studies	Prevalence (%)	95% CI	*I^2^* (%)	*Q*	Heterogeneity test	
						DF	*P*
Blood (bloodstream VRE)	54	15.9	12.0–20.8	97.19	1883.325	53	<0.001
Stool (VRE colonization/carriage)	34	20.7	16.1–26.2	96.11	848.256	33	<0.001
Rectal swab (VRE colonization/carriage)	32	19.9	14.3–27.0	98.15	1672.286	31	<0.001

Country	Number of studies	Prevalence (%)	95% CI	*I^2^* (%)	*Q*	Heterogeneity test	
						DF	*P*
USA	30	22.1	15.9–29.9	98.65	2153.598	29	<0.001
Germany	8	16.9	7.8–32.6	98.04	356.746	7	<0.001
Turkiye	9	19.8	12.3–30.3	90.65	85.544	8	<0.001
Serbia	3	32.9	19.2–50.3	78.69	9.384	2	0.009
Italy	9	10.3	5.0–19.8	97.88	376.752	8	<0.001
Canada	1	1.6	0.1–21.1	-	-	-	-
India	5	16.4	9.5–26.8	77.29	17.610	4	0.001
Iran	3	13.9	4.1–38.1	90.86	21.876	2	<0.001
UK	2	40.1	35.6–44.7	0.00	0.294	1	0.588
Ireland	1	2.9	1.1–7.5	-	-	-	-
Netherlands	2	3.6	0.5–21.5	95.41	21.763	1	<0.001
France	2	31.0	3.2–85.9	98.0	50.063	1	<0.001
Australia	5	22.8	5.5–60.0	97.24	144.890	4	<0.001
Palestine	3	30.0	8.9–66.7	87.99	16.659	2	<0.001
Bulgaria	3	23.2	16.1–32.1	63.51	5.481	2	0.065
South Korea	5	27.5	13.4–48.2	97.16	140.938	4	<0.001
Taiwan	2	32.4	13.0–60.5	96.23	26.509	1	<0.001
Pakistan	1	27.2	22.0–33.1	-	-	-	-
China	3	5.8	3.1–10.5	0.00	0.571	2	0.752
Mexico	2	1.8	0.2–12.3	77.02	4.352	1	0.037
Poland	4	30.8	17.1–48.9	81.02	15.806	3	<0.001
Ethiopia	3	6.0	2.2–15.1	76.44	8.489	2	0.014
Russia	2	18.3	8.2–36.0	96.28	26.880	1	<0.001
Thailand	1	7.5	2.9–18.4	-	-	-	-
Spain	1	6.0	3.2–11.2	-	-	-	-
Iraq	1	50.0	31.7–68.3	-	-	-	-
Saudi Arabia	3	21.1	6.5–50.9	96.01	50.154	2	<0.001
Egypt	1	12.0	5.5–24.2	-	-	-	-
Brazil	2	25.5	6.0–64.5	97.42	38.807	1	<0.001
Qatar	1	22.2	5.6–57.9	-	-	-	-
Greece	1	69.2	49.5–83.8	-	-	-	-
Tunisia	1	0.2	0.1–1.0	-	-	-	-

Continents	Number of studies	Prevalence (%)	95% CI	*I^2^* (%)	*Q*	Heterogeneity test	
						DF	*P*
North America	33	19.3	13.9–26.3	98.57	2240.058	32	<0.001
Europe	47	18.1	14.0–23.1	97.01	1540.216	46	<0.001
Asia	28	21.1	15.5–28.0	94.05	454.104	27	<0.001
Australia	5	22.8	5.5–60.0	97.24	144.890	4	<0.001
Africa	5	3.8	1.2–117	88.23	33.996	4	<0.001
South America	2	25.5	6.0–64.5	97.24	38.807	2	<0.001

Hematological malignancy	Number of studies	Prevalence (%)	95% CI	*I^2^* (%)	*Q*	Heterogeneity test	
						DF	*P*
AML	42	68.3	58.7–76.5	88.01	341.960	41	<0.001
ALL	28	46.3	33.7–59.3	85.85	190.850	27	<0.001
MM	12	10.5	6.4–16.8	50.15	22.067	11	<0.001
NHL	9	11.5	5.7–21.8	69.03	25.832	8	<0.001
CLL	4	7.6	4.0–13.9	0.0	2.857	3	0.414
HSCT	44	97.1	94.1–98.6	90.79	466.876	43	<0.001
Lymphoma	8	16.2	9.8–25.5	60.29	17.626	7	0.014
MDS	11	12.6	8.9–17.7	55.05	22.246	10	0.014
ES	2	6.9	1.7–23.8	0.0	0.003	1	0.960
HL	4	6.5	3.3–12.5	0.0	0.229	3	0.973
HYP	1	6.7	0.9–35.2	-	-	-	-
WAS	1	6.7	0.9–35.2	-	-	-	-
Tumor	6	60.7	15.4–93.0	89.64	48.266	5	<0.001
DLBL	1	7.1	1.0–37.0	-	-	-	-
APML	1	7.1	1.0–37.0	-	-	-	-
Anemia	1	5.9	0.8–32.0	-	-	-	-
Neutropenia	23	91.2	75.7–97.2	79.65	108.095	22	<0.001
CML	4	8.6	2.6–24.8	69.77	9.923	3	0.019
AA	3	4.5	2.2–9.2	0.0	0.286	2	0.867
ARM	1	5.0	0.7–28.2	-	-	-	-
IDS	3	42.0	3.8–92.9	76.92	8.666	2	0.013
SCID	1	7.1	1.0–37.0	-	-	-	-
TC	1	1.7	0.2–10.9	-	-	-	-
OC	1	1.7	0.2–10.9	-	-	-	-
							

Detection method	Number of studies	Prevalence (%)	95% CI	*I^2^* (%)	*Q*	Heterogeneity test	
						DF	*P*
Agar dilution	4	28.2	14.0–48.8	97.45	117.771	3	<0.001
E test	36	22.3	18.4–26.7	93.73	558.444	35	<0.001
Disk diffusion	29	16.8	11.5–24.0	95.61	638.160	28	<0.001
VITEK 2	16	18.5	10.4–30.8	98.44	963.021	15	<0.001
Broth microdilution	13	12.4	7.7–19.4	95.36	258.503	12	<0.001
Microscan	3	42.0	38.3–45.7	9.68	2.214	2	0.331
PCR	7	9.4	2.8–27.4	98.72	470.294	6	<0.001
Chrom agar	6	23.9	9.6–48.3	98.80	416.494	5	<0.001
Phoenix system	5	21.0	2.0–77.8	97.22	143.998	4	<0.001
Sensititre	1	7.5	2.9–18.4	-	-	-	-

AML, acute myeloid leukemia; CML, chronic myeloid leukemia; ALL, acute lymphocytic leukemia; CLL, chronic lymphocytic leukemia; HSCT, hematopoietic stem cell transplant; MM, multiple myeloma; MDS, myelodysplastic syndrome; HL, Hodgkin’s lymphoma; NHL, non-Hodgkin’s lymphoma; AA, aplastic anemia; IDS, immunodeficiency syndrome; SCID, severe combined immunodeficiency syndrome; ES, Ewing’s sarcoma; HYP, hypophosphatasia; WAS, Wiskatt-Aldrich syndrome; ARM, arthritis metothrevat; APML, acute promyelocytic leukemia; DLBL, diffuse large-cell-B-cell lymphoma; USA, United States of America; UK, United Kingdom; PCR, polymerase chain reaction; E test, epsilometer test.

#### 3.3.1 Outcome-specific prevalence of VRE

The overall pooled prevalence of VRE detection among patients with hematological malignancies was 18.4% (95% CI: 15.5–21.6%), with substantial between-study heterogeneity (*I^2^* = 97.56%, *p* < 0.001).

When outcomes were stratified according to clinical context and specimen type, VRE colonization/carriage studies based on stool or rectal-swab specimens demonstrated a higher pooled prevalence than bloodstream VRE detection studies based on blood specimens. The pooled prevalence of VRE was 20.7% among stool-based studies and 19.9% among rectal-swab studies, whereas blood-based studies demonstrated a pooled prevalence of 15.9% (Table 2). The forest plot for this analysis can be found in Supplementary Figure 2.

These findings indicate that gastrointestinal VRE carriage was more frequently detected than bloodstream VRE among the included populations.

The higher prevalence observed in stool and rectal-swab studies could be as a measure of VRE colonization/carriage rather than clinical infection. Conversely, blood-based detection represents a more invasive clinical context; however, because the included studies did not consistently provide standardized clinical criteria for bloodstream infection, these findings are reported as bloodstream VRE detection rather than confirmed VRE bloodstream infection.

#### 3.3.2 Country-level prevalence of VRE in patients with hematological malignancies

Subgroup analysis by 32 countries demonstrated substantial geographic variation in the prevalence of VRE among patients with hematological malignancies. The highest prevalence estimates were observed in Greece (69.2%), Iraq (50.0%), and the United Kingdom (40.1%), whereas the lowest prevalence rates were reported in Tunisia (0.2%), Canada (1.6%), and Mexico (1.8%). Among countries contributing larger numbers of studies, the United States accounted for the highest number of included studies (*n* = 30) and showed a pooled prevalence of 22.1% (95% CI: 15.9–29.9), with considerable heterogeneity (*I^2^* = 98.65%, *p* < 0.001). Similarly, Germany, Türkiye, Italy, South Korea, and Poland demonstrated moderate-to-high pooled prevalence estimates ranging from 10.3 to 30.8%, all with substantial heterogeneity (Table 2).

Several countries, including China and the United Kingdom, exhibited low or no observed heterogeneity despite differing prevalence estimates, suggesting relatively consistent findings across included studies. In contrast, most subgroup analyses demonstrated very high heterogeneity (*I^2^* > 75%), indicating marked variability in study populations, diagnostic methods, sample type and the type of hematological malignancy. Countries represented by a single study did not permit heterogeneity assessment (Table 2). The forest plot for this analysis can be found in Supplementary Figure 3.

#### 3.3.3 Prevalence of VRE in patients with hematological malignancies across continents

Subgroup analysis by continent demonstrated regional variation in the prevalence of VRE among patients with hematological malignancies. South America showed the highest pooled prevalence (25.5%), followed by Australia (22.8%), Asia (21.1%), North America (19.3%), and Europe (18.1%) (Table 2). Africa reported the lowest prevalence estimate at 3.8%. North America and Europe contributed the largest number of studies, with 33 and 47 studies, respectively. Considerable heterogeneity was observed across all continental subgroups, with *I^2^* values exceeding 88%, indicating substantial variability among included studies (Table 2). The forest plot for this analysis can be found in Supplementary Figures 4, 5 shows the continental map of the distribution of VRE in patients with hematological malignancies.

#### 3.3.4 VRE in patients with hematological malignancies in relation to the type of hematological malignancies

Subgroup analysis according to hematological condition demonstrated substantial variation in pooled VRE detection estimates. The highest pooled estimate was observed among studies involving HSCT recipients (97.1, 95% CI: 94.1–98.6%), followed by studies involving neutropenic patients (91.2, 95% CI: 75.7–97.2%) and AML patients (68.3%). These estimates should be interpreted as pooled proportions among the specific study populations included in each subgroup and should not be considered representative estimates of VRE prevalence among all HSCT recipients, neutropenic patients, or AML patients worldwide. The unusually high estimates may reflect the selection of highly immunocompromised, hospitalized, or actively screened populations, as well as differences in study-specific denominators, surveillance strategies, specimen types, and outcome definitions. Other subgroups demonstrated lower pooled estimates, including ALL (46.3%), lymphoma (16.2%), MDS (12.6%), MM (10.5%), NHL (11.5%), CLL (7.6%), CML (8.6%), AA (4.5%), and HL (6.5%). Considerable between-study heterogeneity was observed across most clinical subgroups (Table 2). The forest plot for this analysis can be found in Supplementary Figure 5.

#### 3.3.5 VRE in patients with hematological malignancies in relation to the detection method

Subgroup analysis based on detection methods showed notable variation in the prevalence of VRE, influenced by both the number of included studies and the degree of heterogeneity. The E-test method contributed the highest number of studies (*n* = 36) and demonstrated a pooled prevalence of 22.3% with substantial heterogeneity (*I^2^* = 93.73%, *p* < 0.001). Similarly, disk diffusion (*n* = 29) and VITEK 2 (*n* = 16) showed moderate prevalence estimates of 16.8 and 18.5%, respectively, both with very high heterogeneity. PCR-based studies (*n* = 7) reported a comparatively lower prevalence of 9.4% but also exhibited extreme heterogeneity (I^2^ = 98.72%). The Microscan system, despite including only three studies, reported the highest prevalence (42.0%) with minimal heterogeneity (*I^2^* = 9.68%, *p* = 0.331), indicating consistent findings across studies (Table 2). The forest plot for this analysis can be found in Supplementary Figure 6.

### 3.4 Meta regression

Meta-regression analysis was performed to explore potential sources of heterogeneity across studies. A random-effects meta-regression model was applied using the methods of moments as the computational option to estimate between-study variance (*τ^2^*). Covariates examined included the continents where the study was carried out, detection method of VRE, and the type of sample collected. Regression coefficients (β) with corresponding standard errors and *p*-values were calculated to assess the strength and significance of associations between study-level variables and reported prevalence. A *p*-value <0.05 was considered statistically significant (Figure 5).

**Figure 5:**
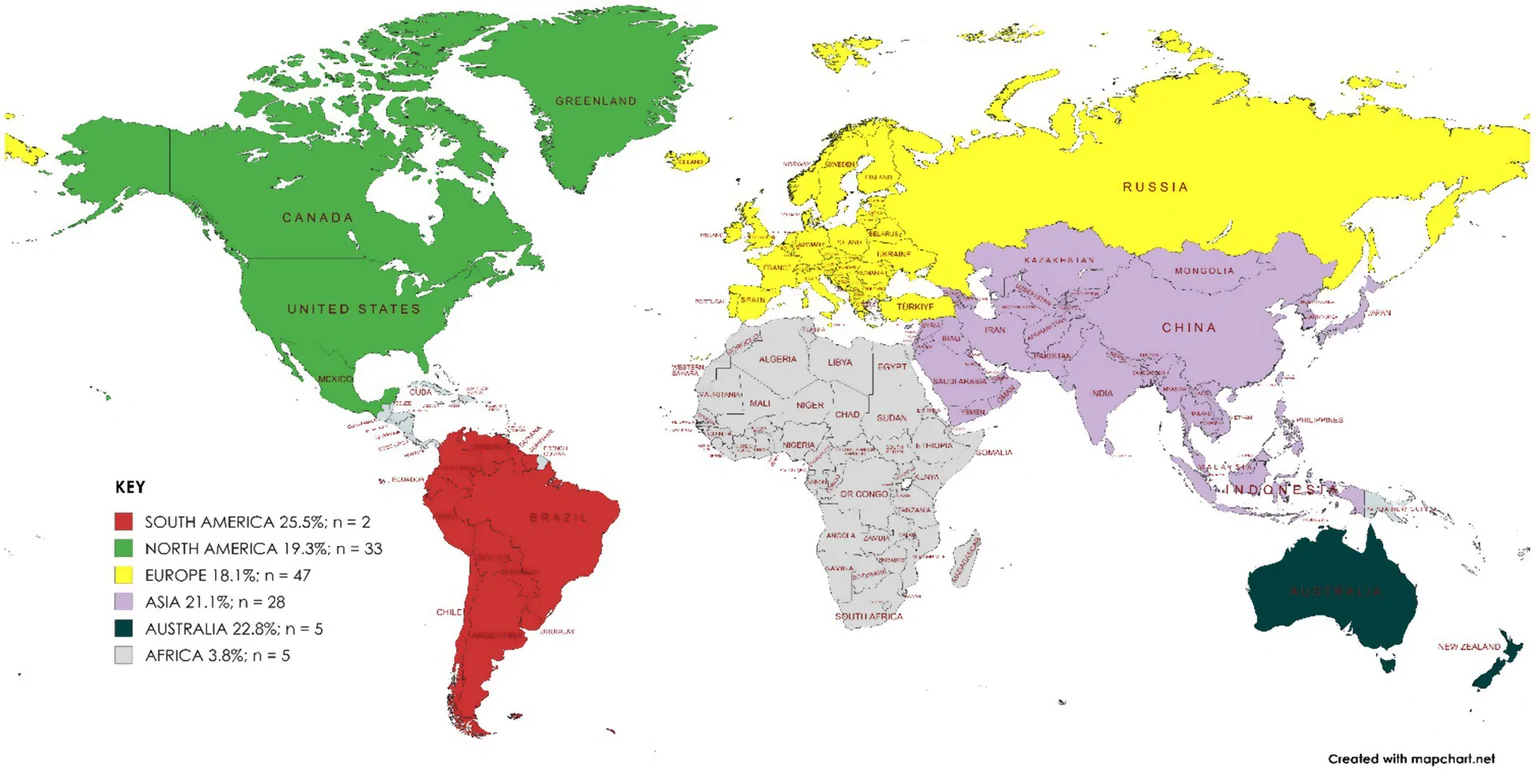
A continental map showing the number of studies and prevalence of VRE in patients with hematological malignancies.

The meta-regression analysis was performed to evaluate whether continent significantly contributed to the heterogeneity observed in the prevalence of VRE among patients with hematological malignancies. Using a random-effects model with logit event rates, continent was identified as a significant moderator of VRE prevalence. Compared with the reference category represented by the intercept, all included continents showed statistically significant positive coefficients, indicating higher log odds of VRE prevalence across regions (Figure 6). The omnibus test for moderators was statistically significant (*Q* = 11.68, *df* = 5, *p* = 0.0395), indicating that continent significantly explained part of the between-study heterogeneity (Figure 6).

**Figure 6:**
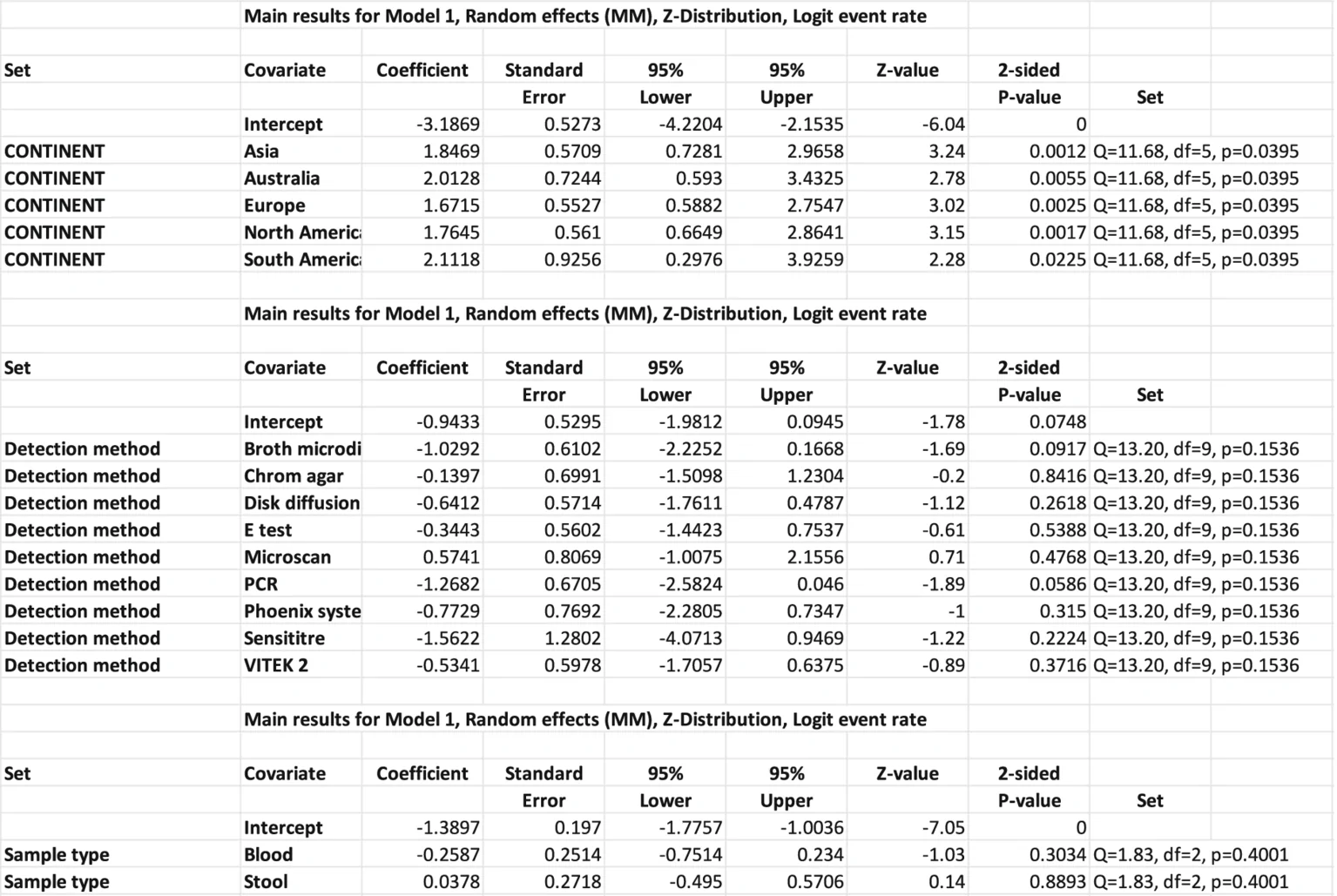
A Meta regression analysis for in relation to the prevalence of VRE in patients with hematological malignancies.

The meta-regression analysis was also conducted to determine whether the detection method significantly contributed to the heterogeneity in the prevalence of VRE among patients with hematological malignancies. Overall, the omnibus test for moderators was not statistically significant (*Q* = 13.20, *df* = 9, *p* = 0.1536), indicating that differences in detection methods did not significantly explain the between-study heterogeneity observed in the meta-analysis (Figure 6).

Further, meta-regression analysis was evaluated to determine whether sample type contributed to the heterogeneity in the prevalence of VRE among patients with hematological malignancies. The overall moderator test was not statistically significant (*Q* = 1.83, *df* = 2, *p* = 0.4001), indicating that differences in sample type did not significantly explain the between-study heterogeneity observed in the meta-analysis (Figure 6).

The corresponding scatterplots for these meta-regression models are presented in Supplementary Figures 7–9.

## 4 Discussion

This systematic review and meta-analysis provides comprehensive global evidence on the prevalence of VRE among patients with hematological malignancies. The pooled prevalence of 18.4% demonstrates a substantial burden of VRE in this highly vulnerable patient population and highlights the growing challenge posed by antimicrobial resistance in hematology and transplant settings. The findings further revealed considerable variability across geographic regions, hematological malignancy types, detection methods, and sample types, with substantial between-study heterogeneity observed throughout the analysis. Overall, this study expands current epidemiological knowledge by synthesizing data from 120 studies across multiple continents and provides one of the most extensive evaluations of VRE prevalence among patients with hematological malignancies to date.

The pooled prevalence observed in this study is consistent with the increasing global burden of multidrug-resistant enterococcal infections reported in immunocompromised populations (21). Enterococci, particularly *E. faecium*, have evolved into major healthcare-associated pathogens due to their intrinsic resistance mechanisms, environmental persistence, and capacity to acquire mobile resistance determinants such as *vanA* and *vanB* genes (1, 5). The high pooled prevalence identified in the present analysis reflects the cumulative impact of prolonged hospitalization, recurrent exposure to broad-spectrum antibiotics, chemotherapy-induced mucosal barrier injury, and severe neutropenia among patients with hematological malignancies. These factors collectively create favorable conditions for gastrointestinal colonization and subsequent dissemination of VRE within healthcare settings.

The prevalence estimate obtained in this study is broadly comparable to the systematic review and meta-analysis conducted by Nabavi et al. (12), which specifically evaluated VRE among HSCT recipients. Their study reported a substantial global burden of VRE colonization and infection among HSCT patients and similarly identified considerable geographic variability and high heterogeneity across included studies. However, the present study extends beyond HSCT populations by incorporating a broader spectrum of hematological malignancies, including AML, ALL, lymphoma, MDS, MM, neutropenic patients, and other immunocompromised hematological conditions. Consequently, the current pooled prevalence may provide a more representative estimate of the overall burden of VRE in hematology practice.

One of the most important findings of this meta-analysis was the extremely high heterogeneity observed across studies (*I^2^* = 97.56%). Such high heterogeneity indicates substantial variability in prevalence estimates that cannot be explained by chance alone. Several factors likely contributed to this variability. First, the included studies originated from diverse geographic regions with differing healthcare infrastructures, infection prevention policies, antimicrobial stewardship programs, and antibiotic usage patterns. Countries with higher antibiotic consumption and weaker infection control measures may experience increased VRE transmission within healthcare settings. Second, the studies included different patient populations with varying degrees of immunosuppression, including HSCT recipients, leukemia patients, and neutropenic individuals, each possessing distinct risk profiles for VRE acquisition and colonization.

Differences in diagnostic and laboratory methodologies also likely contributed significantly to the observed heterogeneity. However, in this study, detection method did not contribute to the heterogeneity observed. Studies employed various detection techniques, including E-test, disk diffusion, PCR, VITEK 2, broth microdilution, Microscan, chromogenic agar, Phoenix systems, and agar dilution. These methods differ in sensitivity, specificity, and ability to accurately detect vancomycin resistance. Molecular techniques such as PCR may identify resistance genes even in low bacterial loads, whereas phenotypic methods rely on culture-based growth and susceptibility testing (22). Variability in laboratory protocols, breakpoint interpretation, and antimicrobial susceptibility standards across countries and institutions may therefore have influenced reported prevalence estimates.

Another important source of heterogeneity relates to differences in specimen types used across studies. Although, in this study, sample type did not contribute to the heterogeneity observed. Blood, stool, and rectal swab specimens reflect different clinical scenarios and stages of VRE acquisition. Stool and rectal swab samples are primarily used for colonization surveillance, while blood cultures represent invasive bloodstream infection (23). Colonization rates are generally expected to exceed bloodstream infection rates because gastrointestinal colonization often precedes invasive disease (24). This distinction likely contributed to the varying prevalence estimates identified across subgroup analyses.

The subgroup analysis by continent demonstrated important geographic variation in VRE prevalence. South America exhibited the highest pooled prevalence (25.5%), followed closely by Australia (22.8%) and Asia (21.1%), whereas Africa reported the lowest prevalence (3.8%). The relatively higher prevalence observed in Asia and South America may reflect increased antimicrobial pressure, widespread empirical use of broad-spectrum antibiotics, overcrowding in tertiary healthcare facilities, and variable infection control practices. In contrast, the lower prevalence observed in Africa may partly reflect underreporting, limited surveillance infrastructure, fewer available studies, and reduced diagnostic capacity rather than a truly lower burden of VRE. Similar geographic variability was also reported by Nabavi et al. (12), who observed substantial regional differences among HSCT recipients. The significant meta-regression finding for continent (*Q* = 11.68, *p* = 0.0395) further confirms that geographic region contributed significantly to between-study heterogeneity.

Country-level analysis revealed substantial variability even within continents. Greece demonstrated the highest prevalence estimate (69.2%), followed by Iraq (50.0%) and the United Kingdom (40.1%). In contrast, Tunisia, Canada, and Mexico reported very low prevalence rates. These findings likely reflect differences in healthcare-associated transmission dynamics, antimicrobial stewardship implementation, hospital infection control practices, and surveillance intensity. Countries such as the United States and several European nations contributed the highest numbers of studies and demonstrated persistently high heterogeneity, suggesting variability in patient populations, study settings, and laboratory practices even within developed healthcare systems.

Subgroup analysis according to hematological malignancy type revealed striking differences in VRE prevalence across clinical groups. HSCT recipients demonstrated the highest prevalence (97.1%), followed closely by neutropenic patients (91.2%) and AML patients (68.3%). These findings are biologically plausible because HSCT recipients undergo intensive immunosuppressive conditioning regimens, prolonged neutropenia, mucosal barrier disruption, and extensive antibiotic exposure, all of which strongly predispose to VRE colonization and infection. The results are consistent with previous studies demonstrating that gastrointestinal VRE colonization is highly prevalent among transplant recipients and is an important predictor of subsequent bloodstream infection (12). The exceptionally high pooled VRE detection estimates observed in the HSCT, neutropenia and AML warrant cautious interpretation. These values should not be extrapolated to the broader populations of patients with these conditions because the included studies often represented selected, high-risk clinical populations rather than unselected cohorts. Patients undergoing HSCT or experiencing profound neutropenia are frequently hospitalized for prolonged periods, exposed to broad-spectrum antimicrobial agents, subjected to intensive microbiological surveillance, and affected by mucosal barrier disruption and profound immunosuppression, all of which may increase the likelihood of VRE detection. In addition, differences in study-specific denominators, sampling strategies, and the distinction between colonization and invasive infection may substantially influence the pooled estimates. Therefore, the subgroup results are best interpreted as evidence of a particularly high burden of VRE detection in selected high-risk hematological populations rather than as estimates of population-wide VRE prevalence. The very high overall heterogeneity observed in this review (*I^2^* = 97.56%) further supports cautious interpretation of these subgroup estimates.

Similarly, AML patients often experience prolonged and profound neutropenia following induction chemotherapy, increasing susceptibility to opportunistic infections. The comparatively lower prevalence observed among patients with MM, MDS, NHL, and HL may reflect differences in treatment intensity, hospitalization duration, and antibiotic exposure. Conditions such as CLL and aplastic anemia demonstrated low heterogeneity, possibly because fewer studies with more homogeneous populations were included in these subgroups.

Subgroup analysis according to detection method also demonstrated notable variability in prevalence estimates. The Microscan system reported the highest prevalence (42.0%) with minimal heterogeneity, suggesting relatively consistent detection across included studies. In contrast, PCR-based methods reported lower prevalence estimates despite extremely high heterogeneity. This discrepancy may be related to differences in molecular targets, assay sensitivity, or selective testing strategies employed in various studies. Nevertheless, meta-regression analysis indicated that detection method was not a statistically significant contributor to heterogeneity (*Q* = 13.20, *p* = 0.1536). Although PCR and broth microdilution showed trends toward lower prevalence estimates, these associations did not achieve statistical significance. These findings suggest that while laboratory methods may influence individual prevalence estimates, they do not fully explain the substantial variability observed across studies.

An important consideration in interpreting the pooled prevalence is the distinction between VRE colonization and invasive infection. In the present review, stool and rectal-swab specimens predominantly represented gastrointestinal colonization or carriage, whereas blood specimens represented bloodstream VRE detection. The higher pooled prevalence observed in stool (20.7%) and rectal-swab (19.9%) studies compared with blood-based studies (15.9%) is therefore biologically and clinically plausible, as gastrointestinal colonization commonly precedes invasive VRE disease in immunocompromised patients, particularly those undergoing HSCT or experiencing profound neutropenia. However, bloodstream detection should not automatically be equated with clinically confirmed VRE infection because standardized clinical infection criteria were not consistently reported across the included studies. This distinction is important because pooling these clinically distinct outcomes can contribute to the very high heterogeneity observed in the present analysis (*I^2^* = 97.56%). Future studies using standardized definitions of colonization and invasive infection will allow more precise estimation of the clinical burden of VRE. However, meta-regression analysis indicated that sample type did not significantly explain between-study heterogeneity (*Q* = 1.83, *p* = 0.4001). These findings suggest that although specimen type influences the magnitude of reported prevalence, other factors such as patient population, healthcare setting, and regional antimicrobial practices likely play larger roles in generating heterogeneity.

The presence of publication bias observed in this study further warrants discussion. Funnel plot asymmetry, significant Egger’s regression, and Begg’s test indicated small-study effects and potential publication bias. The Trim-and-Fill analysis identified 13 potentially missing studies and increased the adjusted pooled prevalence estimate from 18.4 to 22.5%, suggesting that the true global burden of VRE among hematological malignancy patients may be even higher than initially estimated. Publication bias may occur because studies reporting high prevalence or statistically significant findings are more likely to be published than studies with negative or low-prevalence results. Additionally, underrepresentation of studies from low-resource settings may contribute to this imbalance. Publication bias tests are less reliable in prevalence meta-analyses with marked heterogeneity.

The findings of this review have important implications for infection prevention and control in hematology, oncology, and HSCT settings, where VRE colonization and infection may have substantial clinical consequences. VRE surveillance policies should be considered an integral component of institutional antimicrobial resistance programmes, particularly in units caring for patients with prolonged hospitalization, intensive antibiotic exposure, neutropenia, and repeated healthcare contact. Active screening for VRE colonization may be particularly relevant in HSCT units and other high-risk oncology wards, where early identification of colonized patients can facilitate targeted infection-control interventions and help reduce opportunities for transmission. However, screening strategies should be adapted to local epidemiology, institutional resources, and the prevalence of VRE rather than applied uniformly across all healthcare settings.

In addition to surveillance, antimicrobial stewardship remains critical because exposure to broad-spectrum antibiotics, particularly agents with activity against anaerobic and Gram-positive flora, can disrupt the intestinal microbiome and create selective pressure favouring VRE colonization and subsequent infection. Antimicrobial stewardship programmes should therefore emphasize appropriate antibiotic selection, dose optimization, and duration of therapy, with de-escalation or discontinuation when clinically justified. These measures may be particularly important in HSCT and oncology populations, in whom repeated antimicrobial exposure is common.

For patients identified as VRE-colonized or infected, appropriate contact precautions and adherence to standard infection prevention practices remain important components of transmission control, particularly during outbreaks or in high-prevalence units. Rigorous hand hygiene, appropriate use of personal protective equipment, dedicated patient-care equipment where feasible, and enhanced environmental cleaning of high-touch surfaces can reduce opportunities for healthcare-associated transmission. Environmental persistence of VRE also highlights the importance of effective cleaning and disinfection protocols, especially in rooms and clinical areas occupied by colonized or infected patients.

For oncology and transplant centres, the findings support a risk-based, multimodal approach combining surveillance, early detection, antimicrobial stewardship, appropriate isolation or contact precautions, and environmental infection-control measures. Such strategies may be especially valuable in HSCT recipients, who have impaired host defenses and may progress from colonization to invasive VRE infection. Importantly, the implementation of VRE prevention strategies should be guided by local resistance patterns, institutional transmission data, patient risk profiles, and available resources. Our findings therefore provide evidence supporting continued attention to VRE as an important infection prevention priority in high-risk hematology and transplant populations. Furthermore, the emergence of daptomycin-resistant and linezolid-resistant enterococci poses additional therapeutic challenges and highlights the need for continued development of novel antimicrobial agents (25).

Despite its strengths, this study has several limitations. First, the extremely high heterogeneity across studies limits the interpretability of pooled prevalence estimates and suggests that findings should be interpreted cautiously. Although subgroup analyses and meta-regression identified continent as a significant contributor to heterogeneity, substantial unexplained variability remained. Second, many included studies were retrospective or single-center investigations, potentially limiting generalizability. Third, differences in study design, patient populations, antimicrobial exposure, hospitalization duration, and infection control practices may have influenced prevalence estimates but could not be fully assessed due to inconsistent reporting across studies.

Another limitation relates to variability in diagnostic methods and susceptibility testing standards across included studies. Some studies used molecular assays, while others relied on phenotypic culture-based methods, potentially introducing measurement bias. Additionally, several countries were represented by only one or two studies, limiting the robustness of subgroup estimates for these regions. The exclusion of non-English publications may also have introduced language bias. Furthermore, the analysis combined studies reporting colonization and bloodstream infection, which represent clinically distinct outcomes with potentially different epidemiological patterns. Original literatures included clinically heterogeneous outcomes and the absence of standardized clinical definitions of invasive VRE infection prevented a more definitive infection-specific meta-analysis.

Nonetheless, this systematic review and meta-analysis provides important global epidemiological evidence regarding VRE among patients with hematological malignancies. The findings demonstrate a substantial burden of VRE worldwide, particularly among HSCT recipients, AML patients, and neutropenic populations. Geographic region was identified as a significant contributor to heterogeneity, while detection method and sample type did not significantly explain between-study variability. Future multicenter prospective studies using standardized diagnostic protocols and harmonized surveillance approaches are needed to improve comparability across regions, adapt them to local context and better define risk factors associated with VRE acquisition and infection in hematological malignancy patients.

## 5 Conclusion

This study highlights the significant global burden of VRE among patients with hematological malignancies and emphasizes the critical importance of surveillance, infection prevention, and antimicrobial stewardship interventions in hematology and transplant settings. The substantial heterogeneity observed across studies reflects the complex interplay of geographic, methodological, and clinical factors influencing VRE epidemiology. Strengthened international surveillance and standardized reporting are urgently needed to guide effective infection control policies and improve clinical outcomes among vulnerable hematological populations.

## Data Availability

The original contributions presented in the study are included in the article/supplementary material, further inquiries can be directed to the corresponding author/s.
