# Supplementary material for: Global prevalence of vancomycin-resistant Enterococcus in patients with haematological malignancies: a systematic review and meta-analysis
Source: Front Public Health. 2026 Oct 15;14:1945305. doi: 10.3389/fpubh.2026.1945305 (PMC13716793; doi:10.3389/fpubh.2026.1945305)
Supplement: Supplementary file 2 [file Supplementary_file_2.docx]

**SUPPLEMENTARY FILE S2:** The quality of the 120 included studies

| **Study name** | | **Checklist*** | | | | | | | | |
| --- | --- | --- | --- | --- | --- | --- | --- | --- | --- | --- |
|  |  | 1 | 2 | 3 | 4 | 5 | 6 | 7 | 8 | 9 |
| 1 | (Suntharam et al., 2002) | Yes | No | Yes | Yes | Yes | Yes | Yes | Yes | Unclear |
| 2 | (Liss et al., 2012) | Yes | No | Yes | Yes | Yes | Yes | Yes | Yes | Yes |
| 3 | (Gedik et al., 2014) | Yes | No | Yes | Yes | Yes | Yes | Yes | Yes | Yes |
| 4 | (Hefazi et al., 2016) | Yes | No | Yes | Yes | Yes | Yes | Yes | Yes | Yes |
| 5 | (Mioljevi et al., 2013) | Yes | No | Yes | Yes | Yes | Yes | Yes | Yes | Yes |
| 6 | (Chhatwal et al., 2020) | Yes | No | Yes | Yes | Yes | Yes | Yes | Yes | Yes |
| 7 | (C. Cattaneo et al., 2018) | Yes | No | Yes | Yes | Yes | Yes | Yes | Yes | Yes |
| 8 | (Ford et al., 2015) | Yes | No | Yes | Yes | Yes | Yes | Yes | Yes | Yes |
| 9 | (Ford et al., 2015) | Yes | No | Yes | Yes | Yes | Yes | Yes | Yes | Yes |
| 10 | (Taur et al., 2012) | Yes | No | Yes | Yes | Yes | Yes | Yes | Yes | Yes |
| 11 | (Kamboj et al., 2010) | Yes | No | Yes | Yes | Yes | Yes | Yes | Yes | Yes |
| 12 | (Calderwood et al., 2008) | Yes | No | Yes | Yes | Yes | Yes | Yes | Yes | Yes |
| 13 | (Weinstock et al., 2007) | Yes | No | Yes | Yes | Yes | Yes | Yes | Yes | Yes |
| 14 | (Matar et al., 2006) | Yes | No | Yes | Yes | Yes | Yes | Yes | Yes | Yes |
| 15 | (Tsiatis et al., 2004) | Yes | No | Yes | Yes | Yes | Yes | Yes | Yes | Yes |
| 16 | (Shaikh et al., 2002) | Yes | No | Yes | Yes | Yes | Yes | Yes | Yes | Yes |
| 17 | (Henning et al., 1996) | Yes | No | Yes | Yes | Yes | Yes | Yes | Yes | Yes |
| 18 | (Montecalvo et al., 1995) | Yes | No | Yes | Yes | Yes | Yes | Yes | Yes | Yes |
| 19 | (Rubin et al., 1992) | Yes | No | Yes | Yes | Yes | Yes | Yes | Yes | Yes |
| 20 | (Levitt et al., 2014) | Yes | No | Yes | Yes | Yes | Yes | Yes | Yes | Yes |
| 21 | (Song et al., 2016) | Yes | No | Yes | Yes | Yes | Yes | Yes | Yes | Yes |
| 22 | (Zirakzadeh et al., 2007) | Yes | No | Yes | Yes | Yes | Yes | Yes | Yes | Yes |
| 23 | (Embil et al., 1996) | Yes | No | Yes | Yes | Yes | Yes | Yes | Yes | Yes |
| 24 | (Thacker et al., 2014a) | Yes | No | Yes | Yes | Yes | Yes | Yes | Yes | Yes |
| 25 | (Nateghian et al., 2011) | Yes | No | Yes | Yes | Yes | Yes | Yes | Yes | Yes |
| 26 | (Aktürk et al., 2016) | Yes | No | Yes | Yes | Yes | Yes | Yes | Yes | Yes |
| 27 | (Devrim et al., 2015) | Yes | No | Yes | Yes | Yes | Yes | Yes | Yes | Yes |
| 28 | (Bradley et al., 2002) | Yes | No | Yes | Yes | Yes | Yes | Yes | Yes | Yes |
| 29 | (Gray & George, 2000) | Yes | No | Yes | Yes | Yes | Yes | Yes | Yes | Yes |
| 31 | (Nourse et al., 2000) | Yes | No | Yes | Yes | Yes | Yes | Yes | Yes | Yes |
| 32 | (Guiot et al., 1991) | Yes | No | Yes | Yes | Yes | Yes | Yes | Yes | Yes |
| 33 | (Schuster et al., 1998) | Yes | No | Yes | Yes | Yes | Yes | Yes | Yes | Yes |
| 34 | (Plessis et al., 1995) | Yes | No | Yes | Yes | Yes | Yes | Yes | Yes | Yes |
| 35 | (Bell et al., 2013) | Yes | No | Yes | Yes | Yes | Yes | Yes | Yes | Unclear |
| 36 | (Padiglione et al., 2003) | Yes | No | Yes | Yes | Yes | Yes | Yes | Yes | Yes |
| 37 | (Arman et al., 2022) | Yes | No | Yes | Yes | Yes | Yes | Yes | Yes | Yes |
| 38 | (Stoeva et al., 2025) | Yes | No | Yes | Yes | Yes | Yes | Yes | Yes | Yes |
| 39 | (Xie et al., 2020) | Yes | No | Yes | Yes | Yes | Yes | Yes | Yes | Yes |
| 40 | (Worth et al., 2007) | Yes | No | Yes | Yes | Yes | Yes | Yes | Yes | Yes |
| 41 | (Roghmann et al., 1997) | Yes | No | Yes | Yes | Yes | Yes | Yes | Yes | Yes |
| 42 | (Oh et al., 2004) | Yes | No | Yes | Yes | Yes | Yes | Yes | Yes | Yes |
| 43 | (Gambarotto et al., 2000) | Yes | No | Yes | Yes | Yes | Yes | Yes | Yes | Yes |
| 44 | (Carvalho et al., 2020) | Yes | No | Yes | Yes | Yes | Yes | Yes | Yes | Yes |
| 45 | (Niyazi et al., 2024) | Yes | No | Yes | Yes | Yes | Yes | Yes | Yes | Yes |
| 46 | (Webb et al., 2020) | Yes | No | Yes | Yes | Yes | Yes | Yes | Yes | Yes |
| 47 | (Kirby et al., 2006) | Yes | No | Yes | Yes | Yes | Yes | Yes | Yes | Yes |
| 48 | (Hristova et al., 2023) | Yes | No | Yes | Yes | Yes | Yes | Yes | Yes | Yes |
| 49 | (Ümit et al., 2016) | Yes | No | Yes | Yes | Yes | Yes | Yes | Yes | Yes |
| 50 | (C. Y. Chen et al., 2017) | Yes | No | Yes | Yes | Yes | Yes | Yes | Yes | Yes |
| 51 | (Ö. Kara et al., 2015) | Yes | No | Yes | Yes | Yes | Yes | Yes | Yes | Yes |
| 52 | (Bajpai et al., 2023) | Yes | No | Yes | Yes | Yes | Yes | Yes | Yes | Unclear |
| 53 | (Choi et al., 2022) | Yes | No | Yes | Yes | Yes | Yes | Yes | Yes | Yes |
| 54 | (Krull et al., 2016) | Yes | No | Yes | Yes | Yes | Yes | Yes | Yes | Yes |
| 55 | (Sabir et al., 2021) | Yes | No | Yes | Yes | Yes | Yes | Yes | Yes | Yes |
| 56 | (S. Chen et al., 2021) | Yes | No | Yes | Yes | Yes | Yes | Yes | Yes | Yes |
| 57 | (Heisel et al., 2017) | Yes | No | Yes | Yes | Yes | Yes | Yes | Yes | Yes |
| 58 | (Alatorre-Fernández et al., 2017) | Yes | No | Yes | Yes | Yes | Yes | Yes | Yes | Yes |
| 59 | (Ozorowski et al., 2009) | Yes | No | Yes | Yes | Yes | Yes | Yes | Yes | Yes |
| 60 | (Mekonnen et al., 2025) | Yes | No | Yes | Yes | Yes | Yes | Yes | Yes | Yes |
| 61 | (Kirdar et al., 2010) | Yes | No | Yes | Yes | Yes | Yes | Yes | Yes | Yes |
| 62 | (Klyasova et al., 2018) | Yes | No | Yes | Yes | Yes | Yes | Yes | Yes | Yes |
| 63 | (Rattanon et al., 2025) | Yes | No | Yes | Yes | Yes | Yes | Yes | Yes | Yes |
| 64 | (Fedorova et al., 2025) | Yes | No | Yes | Yes | Yes | Yes | Yes | Yes | Yes |
| 65 | (Leis et al., 2006) | Yes | No | Yes | Yes | Yes | Yes | Yes | Yes | Yes |
| 66 | (Torres et al., 2022) | Yes | No | Yes | Yes | Yes | Yes | Yes | Yes | Yes |
| 67 | (Braak et al., 2000) | Yes | No | Yes | Yes | Yes | Yes | Yes | Yes | Yes |
| 68 | (Sengar et al., 2014) | Yes | No | Yes | Yes | Yes | Yes | Yes | Yes | Yes |
| 69 | (Wang et al., 2015) | Yes | No | Yes | Yes | Yes | Yes | Yes | Yes | Yes |
| 70 | (de la Cruz-Hernández et al., 2020) | Yes | No | Yes | Yes | Yes | Yes | Yes | Yes | Yes |
| 71 | (Mattei et al., 2022) | Yes | No | Yes | Yes | Yes | Yes | Yes | Yes | Yes |
| 72 | (Kaveh et al., 2016) | Yes | No | Yes | Yes | Yes | Yes | Yes | Yes | Yes |
| 73 | (Ahmad et al., 2024) | Yes | No | Yes | Yes | Yes | Yes | Yes | Yes | Yes |
| 74 | (Chiara Cattaneo et al., 2008) | Yes | No | Yes | Yes | Yes | Yes | Yes | Yes | Yes |
| 75 | (Hackney et al., 2019) | Yes | No | Yes | Yes | Yes | Yes | Yes | Yes | Yes |
| 76 | (S. Ali et al., 2018) | Yes | No | Yes | Yes | Yes | Yes | Yes | Yes | Yes |
| 77 | (A. Kara et al., 2015) | Yes | No | Yes | Yes | Yes | Yes | Yes | Yes | Yes |
| 78 | (Hung et al., 2021) | Yes | No | Yes | Yes | Yes | Yes | Yes | Yes | Yes |
| 79 | (Amanati et al., 2021) | Yes | No | Yes | Yes | Yes | Yes | Yes | Yes | Yes |
| 80 | (Knoll et al., 2005) | Yes | No | Yes | Yes | Yes | Yes | Yes | Yes | Yes |
| 81 | (Scheich et al., 2018) | Yes | No | Yes | Yes | Yes | Yes | Yes | Yes | Yes |
| 82 | (Satlin et al., 2014) | Yes | No | Yes | Yes | Yes | Yes | Yes | Yes | Yes |
| 83 | (Joudeh et al., 2023) | Yes | No | Yes | Yes | Yes | Yes | Yes | Yes | Yes |
| 84 | (MacAllister et al., 2018) | Yes | No | Yes | Yes | Yes | Yes | Yes | Yes | Yes |
| 85 | (Teferi et al., 2025) | Yes | No | Yes | Yes | Yes | Yes | Yes | Yes | Unclear |
| 86 | (ÇALIŞKAN DEMİRKIRAN et al., 2026) | Yes | No | Yes | Yes | Yes | Yes | Yes | Yes | Yes |
| 87 | (Khateb et al., 2025) | Yes | No | Yes | Yes | Yes | Yes | Yes | Yes | Yes |
| 88 | (M. Ali et al., 2006) | Yes | No | Yes | Yes | Yes | Yes | Yes | Yes | Yes |
| 89 | (Kim & Choi, 2022) | Yes | No | Yes | Yes | Yes | Yes | Yes | Yes | Yes |
| 90 | (Mendes et al., 2020) | Yes | No | Yes | Yes | Yes | Yes | Yes | Yes | Yes |
| 91 | (Park et al., 2024) | Yes | No | Yes | Yes | Yes | Yes | Yes | Yes | Yes |
| 92 | (Yu et al., 2015) | Yes | No | Yes | Yes | Yes | Yes | Yes | Yes | Yes |
| 93 | (Ornstein et al., 2015) | Yes | No | Yes | Yes | Yes | Yes | Yes | Yes | Yes |
| 94 | (Sadowska-Klasa et al., 2017) | Yes | No | Yes | Yes | Yes | Yes | Yes | Yes | Yes |
| 95 | (Battipaglia et al., 2019) | Yes | No | Yes | Yes | Yes | Yes | Yes | Yes | Yes |
| 96 | (Vydra et al., 2012) | Yes | No | Yes | Yes | Yes | Yes | Yes | Yes | Yes |
| 97 | (Dragičević-Jojkić et al., 2021) | Yes | No | Yes | Yes | Yes | Yes | Yes | Yes | Yes |
| 98 | (Navarra et al., 2025) | Yes | No | Yes | Yes | Yes | Yes | Yes | Yes | Yes |
| 99 | (Fanci et al., 1999) | Yes | No | Yes | Yes | Yes | Yes | Yes | Yes | Yes |
| 100 | (Zajac-Spychala et al., 2021) | Yes | No | Yes | Yes | Yes | Yes | Yes | Yes | Yes |
| 101 | (El Omri et al., 2024) | Yes | No | Yes | Yes | Yes | Yes | Yes | Yes | Yes |
| 102 | (Mokhtar et al., 2025) | Yes | No | Yes | Yes | Yes | Yes | Yes | Yes | Yes |
| 103 | (Baier et al., 2019) | Yes | No | Yes | Yes | Yes | Yes | Yes | Yes | Yes |
| 104 | (Papanicolaou et al., 2019) | Yes | No | Yes | Yes | Yes | Yes | Yes | Yes | Yes |
| 105 | (Iosifidis et al., 2012) | Yes | No | Yes | Yes | Yes | Yes | Yes | Yes | Yes |
| 106 | (Dubberke et al., 2006) | Yes | No | Yes | Yes | Yes | Yes | Yes | Yes | Yes |
| 107 | (Alotaibi & Bukhari, 2017) | Yes | No | Yes | Yes | Yes | Yes | Yes | Yes | Yes |
| 108 | (Marchi et al., 2018) | Yes | No | Yes | Yes | Yes | Yes | Yes | Yes | Unclear |
| 109 | (Thacker et al., 2014b) | Yes | No | Yes | Yes | Yes | Yes | Yes | Yes | Yes |
| 110 | (Mandal et al., 2024) | Yes | No | Yes | Yes | Yes | Yes | Yes | Yes | Yes |
| 111 | (Hijazi et al., 2009) | Yes | No | Yes | Yes | Yes | Yes | Yes | Yes | Yes |
| 112 | (Mellouli et al., 2021) | Yes | No | Yes | Yes | Yes | Yes | Yes | Yes | Yes |
| 113 | (Doganci et al., 2023) | Yes | No | Yes | Yes | Yes | Yes | Yes | Yes | Yes |
| 114 | (Nowicka & Gil, 2026) | Yes | No | Yes | Yes | Yes | Yes | Yes | Yes | Yes |
| 115 | (Colaneri et al., 2025) | Yes | No | Yes | Yes | Yes | Yes | Yes | Yes | Yes |
| 116 | (Guarnera et al., 2023) | Yes | No | Yes | Yes | Yes | Yes | Yes | Yes | Yes |
| 117 | (Janjusevic et al., 2021) | Yes | No | Yes | Yes | Yes | Yes | Yes | Yes | Yes |
| 118 | (Cento et al., 2025) | Yes | No | Yes | Yes | Yes | Yes | Yes | Yes | Yes |
| 119 | (Kampmeier et al., 2017) | Yes | No | Yes | Yes | Yes | Yes | Yes | Yes | Yes |
| 120 | (Kim et al., 2003) | Yes | No | Yes | Yes | Yes | Yes | Yes | Yes | Yes |

|  | (Thomas et al., 2007) |
| --- | --- |

*** 1.** Appropriate sampling frame to address target population, **2.** Appropriate sampling way of study participants, **3.** Adequate sample size, **4.** Detail description of study participants and settings, **5.** Data analysis with sufficient coverage of identified sample, **6.** Use of valid methods to identify the condition, **7.** Standard, reliable way of measurement of condition for all participants, **8.** Availability of appropriate statistical analysis, **9.** Adequate response rate and management of low response rate

**Quality of included studies by JBI critical appraisal checklist for studies reporting prevalence data**

*** 1.** Appropriate sampling frame to address target population, **2.** Appropriate sampling way of study participants, **3.** Adequate sample size, **4.** Detail description of study participants and settings, **5.** Data analysis with sufficient coverage of identified sample, **6.** Use of valid methods to identify the condition, **7.** Standard, reliable way of measurement of condition for all participants, **8.** Availability of appropriate statistical analysis, **9.** Adequate response rate and management of low response rate

**Quality of included studies by JBI critical appraisal checklist for studies reporting prevalence of VRE in patients with hematological malignancies**
