# Supplementary material for: Global prevalence of vancomycin-resistant Enterococcus in patients with haematological malignancies: a systematic review and meta-analysis
Source: Front Public Health. 2026 Oct 15;14:1945305. doi: 10.3389/fpubh.2026.1945305 (PMC13716793; doi:10.3389/fpubh.2026.1945305)
Supplement: Supplementary file 3 [file Supplementary_file_3.docx]

**Supplementary Figure 1:** Leave-one-out forest plot of the prevalence of VRE in patients with hematological malignancies

**Supplementary Figure 2**: Forest plot of prevalence of VRE in patients of hematological malignancies in relation to the sample type.

**Supplementary Figure 3:** Forest plot of the country-level prevalence of of VRE in patients with hematological malignancies

**Supplementary Figure 4**: Forest plot of the continent-level prevalence of of VRE in patients with hematological malignancies

**Supplementary Figure 5:** Forest plot of prevalence VRE in patients with hematological malignancies in relation to the type of hematological malignancies

**Supplementary Figure 6:** Forest plot of prevalence of VRE in patients of hematological malignancies in relation to the detection method

**Supplementary Figure 7:** Scatter plot of meta regression on continent variable

**Supplementary Figure 8:** Scatter plot of meta regression on detection method variable

**Supplementary Figure 9:** Scatter plot of meta regression on sample type variable
