# Supplementary material for: Global prevalence of vancomycin-resistant Enterococcus in patients with haematological malignancies: a systematic review and meta-analysis
Source: Front Public Health. 2026 Oct 15;14:1945305. doi: 10.3389/fpubh.2026.1945305 (PMC13716793; doi:10.3389/fpubh.2026.1945305)
Supplement: Supplementary file 4 [file Table_1.docx]

**Supplementary Table 1:** Characteristics of Hematological malignancies as reported by different studies

| **S/NO** | **Author, Publication year** | **Haematological Malignancies** | **Sample Size** | **Number positive** |
| --- | --- | --- | --- | --- |
| 1 | Suntharam et al., 2002 | AML | 12 | 12 |
| 2 | Liss et al., 2012 | AML | 51 | 51 |
| 3 | Gedik et al. 2014 | AML | 50 | 39 |
| 4 | Gedik et al.., 2014 | ALL | 50 | 9 |
| 5 | Gedik et al.,, 2014 | MM | 50 | 1 |
| 6 | Gedik et al., 2014 | NHL | 50 | 1 |
| 7 | Hefazi et a., 2016 | AML | 96 | 96 |
| 8 | Mioljevic et al.,, 2013 | ALL | 27 | 5 |
| 9 | Mioljevic et al.,. 2013 | CLL | 27 | 2 |
| 10 | Mioljevic et al., 2013 | NHL | 27 | 1 |
| 11 | Mioljevic et al. 2013 | AML | 27 | 19 |
| 12 | Chatawal et al., 2020 | HSCT | 132 | 132 |
| 13 | Cattaneo et al. 2018 | AML | 9 | 4 |
| 14 | Cattaneo et al., 2018 | Lymphoma | 9 | 5 |
| 15 | Ford et al., 2015 | HSCT | 224 | 224 |
| 16 | Ford 2015 | AML | 82 | 61 |
| 17 | Ford, 2015 | ALL | 82 | 21 |
| 18 | Taur et al., 2012 | AML | 35 | 35 |
| 19 | Kamboj et al., 2010 | HSCT | 68 | 68 |
| 20 | Calderwood et al., 2015 | HSCT | 134 | 134 |
| 21 | Weinstock et al. 2007 | AML | 37 | 37 |
| 22 | Matar et al. 2007 | AML | 99 | 56 |
| 23 | Matar et al.., 2007 | HSCT | 99 | 32 |
| 24 | Matar et al., 2007 | Lymphoma | 99 | 11 |
| 25 | Tsiatis et al. 2004 | AML | 15 | 6 |
| 26 | Tsiatis et al, 2004 | ALL | 15 | 4 |
| 27 | Tsiatis et al.., 2004 | MDS | 15 | 1 |
| 28 | Tsiatis et al.,, 2004 | ES | 15 | 1 |
| 29 | Tsiatis et al.. 2004 | HL | 15 | 1 |
| 30 | Tsiatis et al,, 2004 | HYP | 15 | 1 |
| 31 | Tsiatis et al., 2004 | WAS | 15 | 1 |
| 32 | Shaikh et al., 2002 | AML | 4 | 4 |
| 33 | Henning et al., 1996 | Tumor | 23 | 23 |
| 34 | Montecalvo et al., 1995 | Tumor | 86 | 86 |
| 35 | Rubin et al., 1992 | Tumor | 4 | 4 |
| 36 | Levitt et al. 2014 | AML | 98 | 65 |
| 37 | Levitt et al., 2014 | HSCT | 98 | 33 |
| 38 | Song et al., 2016 | HSCT | 3 | 3 |
| 39 | Zirakzadeh et al. 2008 | AML | 22 | 13 |
| 40 | Zirakzadeh et al, 2008 | MDS | 22 | 4 |
| 41 | Zirakzadeh et al., 2008 | ALL | 22 | 3 |
| 42 | Zirakzadeh et al.., 2008 | CLL | 22 | 1 |
| 43 | Embil et al., 1996 | HSCT | 0 | 0 |
| 44 | Thacker et al., 2014 | HSCT | 65 | 65 |
| 45 | Nateghian et al., 2011 | ALL | 33 | 33 |
| 46 | Akturk et al. 2015 | ALL | 72 | 35 |
| 47 | Akturk et al., 2015 | AML | 72 | 18 |
| 48 | Akturk et al.., 2015 | Tumor | 72 | 19 |
| 49 | Devim et al.,, 2015 | ALL | 24 | 18 |
| 50 | Devim et al., 2015 | AML | 24 | 4 |
| 51 | Devim et al.. 2015 | HL | 24 | 2 |
| 52 | Bradley et al., 2002 | HSCT | 120 | 120 |
| 53 | Gray et al., 2000 | ALL | 59 | 59 |
| 54 | Nourse et al., 2000 | HSCT | 4 | 4 |
| 55 | Guoit et al., 1991 | HSCT | 9 | 9 |
| 56 | Schuster et al., 1998 | HSCT | 24 | 24 |
| 57 | Pleiss et al., 1995 | HSCT | 14 | 14 |
| 58 | Bass et al., 2013 | HSCT | 18 | 18 |
| 59 | Padiglione et al., 1998 | HSCT | 4 | 4 |
| 60 | Arman et al., 2022 | HSCT | 9 | 9 |
| 61 | Stoeva et al., 2025 | HSCT | 45 | 45 |
| 62 | Xie et al., 2020 | AML | 49 | 49 |
| 63 | Worth et al., 2007 | AML | 14 | 9 |
| 64 | Worth et al.. 2007 | DLBL | 14 | 1 |
| 65 | Worth et al,, 2007 | NHL | 14 | 1 |
| 66 | Worth et al.,. 2007 | MM | 14 | 1 |
| 67 | Worth et al. 2007 | ES | 14 | 1 |
| 68 | Worth et al… 2007 | APML | 14 | 1 |
| 69 | Roghmann et al., 1997 | ALL | 33 | 33 |
| 70 | Oh et al. 2004 | AML | 17 | 14 |
| 71 | Oh et al., 2004 | Anemia | 17 | 1 |
| 72 | Oh et al.., 2004 | MM | 17 | 1 |
| 73 | Oh et al.,, 2004 | Lymphoma | 17 | 1 |
| 74 | Gambarotto et al., 2000 | Neutropenia | 44 | 44 |
| 75 | Carvalho et al., 2020 | AML | 25 | 25 |
| 76 | Niyazi et al., 2024 | HSCT | 15 | 15 |
| 77 | Webb et al., 2020 | Neutropenia | 480 | 480 |
| 78 | Kirby et al., 2006 | Neutropenia | 88 | 88 |
| 79 | Hristova et al. 2023 | MDS | 19 | 6 |
| 80 | Hristova et al., 2023 | MM | 19 | 7 |
| 81 | Hristova et al.,, 2023 | CLL | 19 | 3 |
| 82 | Hristova et al.., 2023 | AML | 19 | 2 |
| 83 | Hristova et al, 2023 | NHL | 19 | 1 |
| 84 | Umit et al., 2016 | AML | 66 | 25 |
| 85 | Umit et al. 2016 | ALL | 66 | 6 |
| 86 | Umit et al, 2016 | CLL | 66 | 3 |
| 87 | Umit et al.. 2016 | MM | 66 | 11 |
| 88 | Umit et al,, 2016 | Lymphoma | 66 | 12 |
| 89 | Umit et al.., 2016 | CML | 66 | 1 |
| 90 | Umit et al.,, 2016 | MDS | 66 | 5 |
| 91 | Umit et al.,. 2016 | Tumor | 66 | 2 |
| 92 | Chen et al., 2017 | Neutropenia | 55 | 55 |
| 93 | Kara et al., 2015 | Neutropenia | 32 | 2 |
| 94 | Vijeta et al., 2023 | Neutropenia | 19 | 1 |
| 95 | Choi et al., 2022 | AML | 35 | 4 |
| 96 | Choi et al. 2022 | ALL | 35 | 30 |
| 97 | Choi et al, 2022 | MM | 35 | 1 |
| 98 | Krull et al, 2016 | Lymphoma | 20 | 1 |
| 99 | Krull et al. 2016 | MM | 20 | 1 |
| 100 | Krull et al., 2016 | AML | 20 | 11 |
| 101 | Krull et al.. 2016 | ALL | 20 | 1 |
| 102 | Krull et al,, 2016 | MDS | 20 | 3 |
| 103 | Krull et al.., 2016 | Histiocystosis | 20 | 1 |
| 104 | Krull et al.,, 2016 | AA | 20 | 1 |
| 105 | Krull et al.,. 2016 | ARM | 20 | 1 |
| 106 | Sabir et al., 2021 | HSCT | 67 | 67 |
| 107 | Chen et al., 2021 | Neutropenia | 0 | 0 |
| 108 | Heisel et al. 2017 | AML | 134 | 123 |
| 109 | Heisel et al., 2017 | MDS | 134 | 11 |
| 110 | Alatore Fernandez et al. 2017 | ALL | 23 | 17 |
| 111 | Alatore Fernandez et al., 2017 | Lymphoma | 23 | 5 |
| 112 | Alatore Fernandez et al, 2017 | MDS | 23 | 1 |
| 113 | Ozorowski et al., 2009 | ALL | 1 | 1 |
| 114 | Mekonnen et al., 2025 | Neutropenia | 2 | 2 |
| 115 | Kirdar et al., 2010 | Neutropenia | 12 | 12 |
| 116 | Klyasova et al., 2018 | HSCT | 52 | 52 |
| 117 | Rattanon et al. 2025 | AML | 4 | 3 |
| 118 | Rattanon et al., 2025 | ALL | 4 | 1 |
| 119 | Federova et al., 2025 | HSCT | 465 | 122 |
| 120 | Leis et al. 2006 | ALL | 31 | 16 |
| 121 | Leis et al., 2006 | NHL | 31 | 11 |
| 122 | Leis et al, 2006 | MDS | 31 | 4 |
| 123 | Torres et al., 2022 | AML | 9 | 9 |
| 124 | Van da Braak et al., 2000 | HSCT | 15 | 15 |
| 125 | Sengar et al., 2014 | Neutropenia | 46 | 46 |
| 126 | Ling et al, 2025 | HSCT | 1 | 1 |
| 127 | De la cruz Hernandez et al., 2020 | Neutropenia | 1 | 1 |
| 128 | Mattei et al., 2022 | Neutropenia | 0 | 0 |
| 129 | Kaveh et al, 2016 | ALL | 14 | 6 |
| 130 | Kaveh et al. 2016 | HL | 14 | 1 |
| 131 | Kaveh et al.. 2016 | NHL | 14 | 3 |
| 132 | Kaveh et al., 2016 | MM | 14 | 2 |
| 133 | Kaveh et al,, 2016 | IDS | 14 | 1 |
| 134 | Kaveh et al.,. 2016 | SCID | 14 | 1 |
| 135 | Ahmed et al., 2024 | HSCT | 13 | 13 |
| 136 | Cattaneo et al., 2008 | ALL | 3 | 3 |
| 137 | Hackney et al., 2019 | AML | 42 | 42 |
| 138 | Ali et al., 2018 | IDS | 7 | 7 |
| 139 | Kara et al. 2015 | IDS | 3 | 1 |
| 140 | Kara et al.., 2015 | AML | 3 | 1 |
| 141 | Kara et al 2015 | ALL | 3 | 1 |
| 142 | Hung et al.,. 2021 | Neutropenia | 66 | 66 |
| 143 | Amanati et al., 2021 | Neutropenia | 2 | 2 |
| 144 | Knoll et al., 2005 | Neutropenia | 44 | 44 |
| 145 | Scheich et al., 2018 | AML | 122 | 122 |
| 146 | Satlin et al., 2014 | HSCT | 56 | 56 |
| 147 | Joudeh et al., 2023 | Neutropenia | 2 | 2 |
| 148 | Mac Allister et al., 2018 | HSCT | 90 | 90 |
| 149 | Teferi et al., 2025 | ALL | 11 | 4 |
| 150 | Teferi et al. 2025 | NHL | 11 | 3 |
| 151 | Teferi et al, 2025 | Tumor | 11 | 1 |
| 152 | Teferi et al.. 2025 | AML | 11 | 3 |
| 153 | Caliskan et al., 2026 | HSCT | 27 | 27 |
| 154 | Khateb et al., 2025 | HSCT | 23 | 23 |
| 155 | Ali et al., 2006 | Neutropenia | 6 | 6 |
| 156 | Kim and Choi, 2022 | ALL | 77 | 8 |
| 157 | Kim and Choi., 2022 | AML | 77 | 33 |
| 158 | Kim and Choi,, 2022 | MDS | 77 | 9 |
| 159 | Kim and Choi,. 2022 | AA | 77 | 4 |
| 160 | Kim and Choi.. 2022 | NHL | 77 | 14 |
| 161 | Kim and Choi,., 2022 | MM | 77 | 9 |
| 162 | Mendes et al., 2021 | HSCT | 53 | 53 |
| 163 | Park et al. 2024 | AML | 18 | 12 |
| 164 | Park et al., 2024 | ALL | 18 | 4 |
| 165 | Park et al, 2024 | MM | 18 | 2 |
| 166 | Yu et al. 2015 | ALL | 8 | 5 |
| 167 | Yu et al., 2015 | CML | 8 | 3 |
| 168 | Ornstein et al 2015 | AML | 37 | 37 |
| 169 | Sadowski-Klasa et al., 2017 | HSCT | 37 | 37 |
| 170 | Battiplagia et al. 2019 | AML | 2 | 1 |
| 171 | Battiplagia et al., 2019 | ALL | 2 | 1 |
| 172 | Vydra et al., 2012 | HSCT | 173 | 173 |
| 173 | Jojkic et al., 2021 | Neutropenia | 7 | 7 |
| 174 | Navarra et al., 2025 | HSCT | 1037 | 1037 |
| 175 | Fanci et al., 1999 | AML | 1 | 1 |
| 176 | Olga et al. 2021 | ALL | 71 | 50 |
| 177 | Olga et al., 2021 | AML | 71 | 15 |
| 178 | Olga et al, 2021 | HL | 71 | 4 |
| 179 | Olga et al.. 2021 | NHL | 71 | 2 |
| 180 | Omri et al., 2024 | Neutropenia | 2 | 2 |
| 181 | Mokhtar et al., 2025 | Neutropenia | 67 | 67 |
| 182 | Baier et al., 2019 | HSCT | 4 | 4 |
| 183 | Papanicolaou et al. 2019 | AML | 258 | 207 |
| 184 | Papanicolaou et al., 2019 | MDS | 258 | 51 |
| 185 | Iosifidis et al., 2012 | HSCT | 18 | 18 |
| 186 | Dubberke et al., 2006 | AML | 60 | 32 |
| 187 | Dubberke et al. 2006 | CML | 60 | 5 |
| 188 | Dubberke et al, 2006 | Lymphoma | 60 | 13 |
| 189 | Dubberke et al,, 2006 | MDS | 60 | 4 |
| 190 | Dubberke et al.. 2006 | MM | 60 | 2 |
| 191 | Dubberke et al.,. 2006 | AA | 60 | 2 |
| 192 | Dubberke et al.,, 2006 | TC | 60 | 1 |
| 193 | Dubberke et al.., 2006 | OC | 60 | 1 |
| 194 | Alotaibi and Bukhari, 2017 | AML | 12 | 2 |
| 195 | Alotaibi and Bukhari., 2017 | MM | 12 | 2 |
| 196 | Alotaibi and Bukhari. 2017 | Neutropenia | 12 | 8 |
| 197 | Marchi et al., 2018 | AML | 34 | 22 |
| 198 | Marchi et al, 2018 | ALL | 34 | 9 |
| 199 | Marchi et al. 2018 | CML | 34 | 2 |
| 200 | Marchi et al.,, 2018 | Lymphoma | 34 | 1 |
| 201 | Thacker et al.. 2014 | Neutropenia | 2 | 2 |
| 202 | Mandal et al., 2024 | Neutropenia | 3 | 3 |
| 203 | Hijazi et al., 2009 | HSCT | 19 | 19 |
| 204 | Mellouli et al., 2021 | HSCT | 2 | 2 |
| 205 | Doganci et al., 2023 | ALL | 5 | 5 |
| 206 | Nowicka et al., 2026 | HSCT | 2 | 2 |
| 207 | Colaneri et al., 2025 | HSCT | 304 | 304 |
| 208 | Guarnera et al., 2023 | AML | 8 | 8 |
| 209 | Janjusevic et al., 2021 | HSCT | 28 | 28 |
| 210 | Cento et al., 2025 | HSCT | 7 | 7 |
| 211 | Kampmeier et al., 2017 | HSCT | 12 | 12 |
| 212 | Kim et al., 2003 | HSCT | 230 | 230 |
| 213 | Thomas et al., 2005 | HSCT | 61 | 61 |
